# Lanatoside C decelerates proliferation and induces apoptosis through inhibition of STAT3 and ROS-mediated mitochondrial membrane potential transformation in cholangiocarcinoma

**DOI:** 10.3389/fphar.2023.1098915

**Published:** 2023-06-15

**Authors:** Chao Zhang, Hong-Ying Yang, Long Gao, Ming-Zhen Bai, Wen-Kang Fu, Chong-Fei Huang, Ning-Ning Mi, Hai-Dong Ma, Ya-Wen Lu, Ning-Zu Jiang, Liang Tian, Teng Cai, Yan-Yan Lin, Xing-Xing Zheng, Kun Gao, Jian-Jun Chen, Wen-Bo Meng

**Affiliations:** ^1^ The First Clinical Medical College, Lanzhou University, Lanzhou, Gansu, China; ^2^ Department of Orthopedics, The First Hospital of Lanzhou University, Lanzhou, Gansu, China; ^3^ State Key Laboratory of Applied Organic Chemistry, College of Chemistry and Chemical Engineering, Lanzhou University, Lanzhou, China; ^4^ Department of General Surgery, The First Hospital of Lanzhou University, Lanzhou, Gansu, China; ^5^ Department of Ophthalmology, The Second Hospital of Lanzhou University, Lanzhou, Gansu, China

**Keywords:** Lanatoside C, cholangiocarcinoma, reactive oxygen species, mitochondrial membrane potential, stat3, apoptosis

## Abstract

**Introduction:** The incidence of cholangiocarcinoma (CCA) has increased worldwide in recent years. Given the poor prognosis associated with the current management approach of CCA, new therapeutic agents are warranted to improve the prognosis of this patient population.

**Methods:** In this study, we extracted five cardiac glycosides (CGs) from natural plants: digoxin, lanatoside A, lanatoside C, lanatoside B, and gitoxin. Follow-up experiments were performed to assess the effect of these five extracts on cholangiocarcinoma cells and compounds with the best efficacy were selected. Lanatoside C (Lan C) was selected as the most potent natural extract for subsequent experiments. We explored the potential mechanism underlying the anticancer activity of Lan C on cholangiocarcinoma cells by flow cytometry, western blot, immunofluorescence, transcriptomics sequencing, network pharmacology and *in vivo* experiments.

**Results:** We found that Lan C time-dependently inhibited the growth and induced apoptosis of HuCCT-1 and TFK-1 cholangiocarcinoma cells. Besides Lan C increased the reactive oxygen species (ROS) content in cholangiocarcinoma cells, decreased the mitochondrial membrane potential (MMP) and resulted in apoptosis. Besides, Lan C downregulated the protein expression of STAT3, leading to decreased expression of Bcl-2 and Bcl-xl, increased expression of Bax, activation of caspase-3, and initiation of apoptosis. N-acetyl-L-cysteine (NAC) pretreatment reversed the effect of Lan C. *In vivo*, we found that Lan C inhibited the growth of cholangiocarcinoma xenografts without toxic effects on normal cells. Tumor immunohistochemistry showed that nude mice transplanted with human cholangiocarcinoma cells treated with Lan C exhibited decreased STAT3 expression and increased caspase-9 and caspase-3 expression in tumors, consistent with the *in vitro* results.

**Conclusion:** In summary, our results substantiates that cardiac glycosides have strong anti-CCA effects. Interestingly the biological activity of Lan C provides a new anticancer candidate for the treatment of cholangiocarcinoma.

## Background

Cholangiocarcinoma (CCA) is an epithelial tumor occurring in the intrahepatic or extrahepatic bile duct with bile duct cell differentiation, anatomically divided into intrahepatic cholangiocarcinoma (iCCA), periportal cholangiocarcinoma (pCCA) and distal cholangiocarcinoma (dCCA) (Razumilava and Gores, 2014). Each subtype is associated with different risk factors, molecular pathogenesis, treatment options and prognosis (Kelley et al., 2020). Current evidence suggests that pCCA has a poor prognosis, with a median survival of fewer than 2 years for patients with advanced disease (Rizvi and Gores, 2013). For the three anatomical subtypes, surgical resection and liver transplantation represent the mainstay of treatment (Rodrigues et al., 2021). However, the median 5-year survival rate after CCA resection is only 30%. Liver transplantation as represents a treatment modality indicated only for iCCA and pCCA. According to an international multicenter study, the 5-year survival rate of liver transplantation was 65% for very early-stage iCCA (tumor size less than 2 cm) compared to 45% for the advanced group (tumor size greater than 2 cm). Despite these encouraging results, most patients presented with advanced-stage disease at diagnosis (Mazzaferro et al., 2020). Standard systemic chemotherapy with gemcitabine and cisplatin is indicated is indicated for patients who are not surgical resection or liver transplantation candidates. However, the median survival for this combination chemotherapy regimen is only 11.7 months (Sato et al., 2020). Moreover, no specific targeted molecular therapies have hitherto been approved for CCA. The poor efficacy of conventional chemotherapeutic compounds and the development of their resistance, has boosted interest traditional compounds and their extracts. The antitumor activity of gentian biosides against 10 lung cancer cells was evaluated by *in vitro* experiments. They found that gentian glycosides inhibited tumor cell proliferation and induced apoptosis by regulating the Bax/Caspase-9/Caspase-3 cell pathway (Chen et al., 2017). Besides, it has been established that curculigo saponin I inhibits the proliferation of osteosarcoma cells by inactivating the Wnt/β-catenin pathway, blocks the cell cycle in the G2/M phase, induces apoptosis and inhibits the invasion and migration of osteosarcoma cells (Chang et al., 2017). Besides, curcumol reportedly plays an anticancer effect on cholangiocarcinoma cells by downregulating CDKL3 (Zhang et al., 2018). In addition, a recent study revealed that radix astragali extract is cytotoxic and inhibits cholangiocarcinoma cell proliferation (Wang et al., 2020). An increasing body of evidence from recently published literature suggests that cardiac glycosides are selectively cytotoxic to cancer cells, which has generated interest in their use as anticancer molecules (Hu et al., 2018; Reddy et al., 2019).

It is widely acknowledged that cardiac glycosides are found in *D. lanata* Ehrh (Kreis, 2017). Currently, the pharmaceutical industry still relies on natural resources, and cardiac glycosides of digitalis origin remain a source of cardiac compounds for clinical practice. For example, the cardiac glycosides digoxin and digitalis toxin for the treating congestive heart failure and arrhythmias can be isolated from the leaves of this plant. Compared to other plants of the genus trichoderma, *Digitalis lanata* has a higher content of cardiac glycosides (Ehle et al., 2011). Cardiac glycosides are compounds of natural origin with steroid and glycone fractions in their structure. Interestingly cardiac glycoside analogs can inhibit subunits of the prevalent transmembrane protein Na+/K + - ATPase (Yan and Shapiro, 2016). Overwhelming evidence substantiates that CGs could block lung cancer cells at G0/G1 phase in a dose-dependent manner, thereby inhibiting cancer cell proliferation (Kaushik et al., 2017). A study revealed that targeting FXYD2 by cardiac glycosides could potently block tumor growth in ovarian clear cell carcinoma (Hsu et al., 2016). In another study, cardiac glycosides induced mitotic arrest and apoptosis in colorectal cancer HT-29 cells through HIF-1α and NF-κB-mediated downregulation of Plk1 expression (Xie et al., 2013). Studies on the anti-cancer effects of cardiac glycosides have substantiated that these compounds have heterogeneous effects on different tumors, and their possible mechanisms of action are different (Pratt et al., 2018; Rasheduzzaman et al., 2019). However, the effects of these compounds on CCA have rarely reported in the literature. Therefore, this study was conducted to investigate the inhibitory effects of cardiac glycosides on CCA and the underlying mechanisms in the quest, to find new candidate compounds for the treatment of cholangiocarcinoma.

Herein, we explored the anticancer effects of Lan C on CCA. We revealed that Lan C could inhibit proliferation and induce apoptosis of CCA cells. Besides, we investigated the potential mechanisms of the cytotoxic effects of Lan C and identified the regulatory role of ROS in the apoptotic process. Overall, the present study provides preliminary evidence that Lan C induces apoptosis in CCA cells by affecting ROS expression, thereby regulating changes in mitochondrial membrane potential.

## Methods

### Reagents and cell culture

Compounds (Y1-Y5) were dissolved in dimethyl sulfoxide and stored at −20°C. N-acetyl-L-cysteine (NAC) was purchased from Sigma (St.Louis, MO, United States). Antibodies, including anti-GAPDH, m-IgGκ BP-HRP and mouse anti-rabbit IgG-HRP, were purchased from Santa Cruz Biotechnology (Santa Cruz, CA, United States). Antibodies, including anti-Bcl-2, anti-Bcl-xl, anti-Bax, anti-caspase-3, anti-cleaved-PARP and anti-STAT3, were purchased from Cell Signaling Technology (Danvers, MA, United States). Antibodies, including anti-caspase-9, were purchased from Abcam (Cambridge, MA, United States). FITC Annexin V apoptosis Detection Kit I and Propidium Iodide (PI) were purchased from BD Pharmingen (Franklin Lakes, NJ, United States). Human cholangiocarcinoma cell line HuCCT-1 was purchased from Shanghai Fu Heng Biological, and TFK-1 was purchased from Creative Bioarray in the United States. HuCCT-1 is a well-established intrahepatic cholangiocarcinoma cell line, that causes iCCA, while TFK-1 is an extrahepatic cholangiocarcinoma cell line, that causes pCCA and dCCA. The cells were maintained in RPMI-1640 medium containing 10% fetal bovine serum (FBS) at 37 °C in a humidified incubator with a 5% CO_2_ atmosphere. The NMR spectra were recorded on a Bruker AVANCE NEO-600 spectrometer. Semipreparative HPLC was carried out on a Waters 1,525 with a reversed-phase C18 (150 × 10 mm, 10 μm) column and Waters 2,998 photodiode array detector. Microporous resins, Sephadex LH-20, and silica gel for crude extract or fractions were purchased from Qingdao Marine Chemical Inc.

### Extraction and isolation of compounds Y1-Y5

The leaves of *D. lanata* were collected in September 2016 at Wuhu City, Anhui Province, P. R. China. The plant was authenticated by Dr. Jian-Yin Li from the School of Pharmacy, Lanzhou University. A voucher specimen (accession number: 20160912) was deposited at the School of State Key Laboratory of Applied Organic Chemistry, College of Chemistry and Chemical Engineering, Lanzhou University. The dried leaves of *D. lanata* (15 kg) were extracted with methanol, concentrated under reduced pressure and vacuum dried. The extract (3.5 kg) was partitioned with EtOAc (420 g). Subsequently, the EtOAc fraction was separated with D101 macroporous resin column chromatography and eluted with H_2_O/MeOH system (100:0, 70:30, 50:50,20:80, 0:100 v/v) to give four fractions (Fr.A–Fr.D). Fr.D (80% MeOH/H_2_O) underwent silica gel column chromatography (CH_2_Cl_2_/MeOH (20:1 to 1:1, v/v)) to yield Fr.D1-Fr.D4. Sephadex LH-20 was used to separate Fr.D1–Fr.D4. Compounds Y2 (Lanatoside A, 2.3 g), Y3 (Lanatoside C, 3.5 g) and Y4 (Lanatoside B, 900 mg) were obtained from Fr.D4 by recrystallization at the mixed solution of CH_2_Cl_2_/MeOH. The supernate of Fr.D4 was purified by semipreparative HPLC to obtain compound Y1 (digoxin, 300 mg). Compound Y5 (gitoxin, 850 mg) was crystallized in a solution of CH_2_Cl_2_/MeOH at fraction Fr.D3.

### Toxicity testing

We first determined the structures of five compounds and then treated two cholangiocarcinoma cell lines with these five compounds: HuCCT-1, an intrahepatic cholangiocarcinoma cell line, and TFK-1, an extrahepatic cholangiocarcinoma cell line.

The compounds were diluted to different concentrations and their inhibition rates were determined by CCK-8 method. The concentration of the compound was taken as the abscissa and the inhibition rate as the ordinate. The concentration of a compound with a inhibition rate of 50% is indicated by IC50. The lower the IC50 value, the stronger the inhibitory effect of the compound on tumor cells. Therefore, ideal compounds were selected based on IC50 for follow-up studies. When this compound was selected, wound healing and transwell assays were performed to further verify its effect. At the same time, CCK-8 assay was performed on human intrahepatic bile duct epithelial cell (HIBEpiC) to observe the toxic effects of the selected compounds on normal cells.

### Live-cell imaging

HuCCT-1 and TFK-1 cells were separated into control and experimental groups. The experimental group was treated with Lan C and incubated in a live cell imager for 48 h. The cell growth was observed at 0, 12, 24, and 48 h to assess the effect of Lan C on cholangiocarcinoma cells.

### Cell apoptosis and cycle analysis by flow cytometry

Cholangiocarcinoma cells were treated with Lan C for 48 h; then, the cells were harvested and washed twice with PBS on ice. The washed cell samples were resuspended in 500 μL of binding buffer, and Annexin V and propidium iodide (PI) were double-stained in the binding buffer for 30 min by FACSCalibur flow cytometer to observe cell apoptosis and cycle change. In some experiments, cells were pretreated with 5 mM NAC for 2 h prior to exposure to Lan C, and changes in apoptosis of two types of cholangiocarcinoma cells were analyzed at this time. In addition, we also observed the effect of Lan C on HIBEpiC apoptosis.

### Tunel cell apoptosis analysis

The adherent cholangiocarcinoma cells were cultured for 24 h, and the culture medium containing Lan C was added to the experimental group. After an additional 48 h of incubation, the medium was discarded, washed once with PBS, and fixed with 4% paraformaldehyde for 30 min. After washing once with PBS, PBS containing 0.3% Triton X-100 was added and incubated for 5 min at room temperature. They were washed twice with PBS. 50 μL of TUNEL assay solution was added to the samples and incubated at 37°C for 60 min in the dark. Cells were washed three times with PBS. Nuclei were counterstained with DAPI working solution and incubated at room temperature in the dark for 5 min. Cells were washed three times with PBS. The slides were sealed with an anti-fluorescence quenching blocking solution and observed under a fluorescence microscope.

### Transcriptomics sequencing and data analysis

Human cholangiocarcinoma cells HuCCT-1 were cultured to the logarithmic growth phase and divided into control and experimental groups. Three samples from each group were prepared for the experiment. The experimental group was cultured in a medium containing Lan C (dissolved in DMSO) for 48 h, the control group was cultured for 48 h in a medium containing the same concentration of DMSO. The adherent cells were trypsinized, centrifuged and suspended in PBS, centrifuged again, and the cell precipitate was collected. 1 mL of Trizol lysate was added to the cell precipitate, cells were gently blown evenly, then blown into the EP tube and sealed. The cells were transported in dry ice for sequencing. After rRNA was extracted, the sequencing library was prepared according to Illumina TruSeq RNA sample preparation guidelines (Illumina, San Diego, California, United States). Once the double-stranded cDNA was synthesized, the index connector was connected. After size selection using Agencurt AMPure XP (Beckman), Qubit 2.0 Fluorometer with Qubit dsDNA HS Analysis Kit (Invitrogen, Eugene, OR, United States) and Agilent Bioanalyzer Quantitative and qualitative library (Agilent Technologies, Santa Clara, CA, United States), submit the sample to Illumina HiSeq X-10 (Illumina, San Diego, CA, US) for double-ended sequencing. Quality control and filtering of fastq data were performed using fastq (Chen et al., 2018). Paired-end reads were then mapped to GRCh38 human reference genome using via the Hisat2 tool (version 2.1.1) (Kim et al., 2015). Using the DESeq package (Anders and Huber, 2013) (version 1.8.3) of R software (version 4.1.0), the transcriptome sequencing results were subjected to differential expression analysis according to criteria: |log2 (FoldChange)| > 1 and significant *p*-value < 0.05. To clarify the potential biological significance of the differential genes, we used the topGO package (version 2.24.0) to perform GO enrichment analysis. GO analysis was used to preliminarily understand the cellular localization, molecular functions and enriched biological processes of the differentially expressed genes. Gene function was based on the following categories: Molecular Function (MF), Cellular Component (CC) and Biological Process (BP). In addition, KEGG signal pathway enrichment analysis provides a better understandind of the biological function of genes, signal transduction pathways, *etc.* The genes were blasted onto the KEGG pathways database using the KAAS webserver (Moriya et al., 2007). Significantly enriched pathways were identified with a *p*-value <0.05.

### Network pharmacology

The target genes of Lan C were predicted using SwissTargetPrediction and Comparative Toxicogenomics Database (CTD). The intersection of the target genes and the differentially expressed genes of transcriptome sequencing was obtained. Next, we performed protein-protein interaction network analysis on the intersected genes using the STRING database to analyze the hub genes in the network. Next, the candidate target protein was selected for molecular docking. We downloaded the 2D structures of compounds from the PubChem database (https://pubchem.ncbi.nlm.nih.gov/) and imported them into Chem3D software to minimize energy (MM2 force field) and converted them into 3D structures. Next, using AutoDock tools 1.5.6, the obtained 3D structures were modified by the addition of hydrogens and protonation. We retrieved the 3D structure of the protein from the Protein Data Bank (PDB) (http://www.rcsb.org/), and imported it into the software Pymol and modified it by removing water molecules, co-crystallized ligand and ions. Subsequently, missing hydrogens and Kollman partial charges were added, non-polar hydrogens were merged to their corresponding carbons. AutoDock tools 1.5.6 was used to construct mating pockets of docking. When constructing the mating box, the spacing (angstrom) was set to 1, the center was set on the macromolecule, and the number of points on the x-, y-, and z-dimension were set to make the protein completely covered by the mating box. Autodock tools was used to preprocess the 3D structures of targets and small molecules, Autodock Vina was utilized for docking, and Pymol software was used to visualize the docking results.

### Western blot

Cholangiocarcinoma cells treated with Lan C for 48 h were homogenized in a protein lysis buffer, centrifuged at 12,000 g for 10 min at 4°C to remove debris, and the protein concentration of the whole cell extract was determined using the Bradford protein assay (Bio-Rad, CA, United States). Equal amounts of lysed proteins were separated by SDS-polyacrylamide gel electrophoresis and electroblotted on polyvinylidene fluoride membranes. The membranes were blocked with 5% nonfat milk for 2 h at room temperature in TBST, followed by incubation with specific primary antibodies (including rabbit anti-Bcl-xl (1: 1,000,CST, United States), rabbit anti-Bax (1: 1,000, CST, United States), mouse anti-Bcl-2 (1: 1,000,CST, United States), rabbit anti-caspase-3 (1: 1,000,CST, United States), mouse anti-STAT3 (1: 1,000,CST, United States), rabbit anti-p-STAT3 (1: 1,000,CST, United States) and mouse anti-caspase-9 (1: 500, Abcam, United States), rabbit anti-Cleaved PARP (1:1,000,CST, United States), mouse anti-GAPDH (1:5,000, Proteintech, United States)) in TBST overnight at 4°C. After 3 washes with TBST, the cells were incubated with secondary antibody [HRP-conjugated anti-rabbit or anti-mouse IgG (1: 3,000, CST, United States)] for 1 h, and immunoreactive bands were visualized using an ECL kit, and detected by the ChemiDoc MP Imaging System (Bio-Rad, California, United States). The band intensity of proteins was quantified using ImageJ software (National Institutes of Health, MD, United States), and the relative protein expression was normalized to GAPDH expression. In some experiments, cells were pretreated with 5 mM NAC for 2 h before exposure to Lan C, and the expression of the above proteins in two types of cholangiocarcinoma cells was verified by this method.

### Measurement of reactive oxygen species production

Cells were plated in 6-well plates overnight in a complete medium, and then treated with Lan C-containing medium for 48 h. Cells were stained with 10 μM DCFH-DA (Beyotime, Shanghai, China) for 30 min at 37°C in the dark. Cells were collected and analyzed for fluorescence by immunofluorescence and flow cytometry. In some experiments, cells were pretreated with 5 mM NAC for 2 h prior to exposure to Lan C and analyzed for trends in ROS.

### Mitochondrial membrane potential assay

The changes of JC-1 in cells were detected by flow cytometry to reflect the changes in mitochondrial membrane potential. Cells were plated in 6-well plates overnight in a complete medium. It was then treated with Lan C-containing medium for 48 h. Cells were stained with 10 μM DCFH-DA (Beyotime, Shanghai, China) for 30 min at 37°C in the dark. Cells were collected and analyzed by immunofluorescence and flow cytometry assays. In some experiments, cells were pretreated with 5 mM NAC for 2 h prior to exposure to Lan C and changes in mitochondrial membrane potential were verified by analysis of JC-1.

### *In Vivo* antitumor studies

*In vivo* experiments were performed using athymic nude mice (Balb/c nu, 4–5 weeks, male). All experimental animal protocols were approved by the Ethics Committee of the First Hospital of Lanzhou University. The experimental animals were randomly divided into control group, NAC + Lan C group, and Lan C group, with 6 nude mice in each group. Animals were housed at constant room temperature on a 12-h light/12-h dark cycle and fed standard water and rodent chow. The right forelimb of the mouse was injected subcutaneously with HuCCT-1 cells (5 × 10^6^ cells, 100 μL serum-free RPMI-1640. When the tumor reached 5 mm^3^, the control group was given gavage with 100 µL PBS/d, the Lan C group was given gavage with 40 mg/kg/d and volume of 100 µL, and the NAC + Lan C group was given gavage with 100 mg/kg/d of NAC 2 h before Lan C, the content of DMSO in the three groups was the same. The length (L) and width (W) of the tumor were measured weekly, and the volume of the tumor was calculated (V = 0.5 × L × W^2^). After the experiment, the animals were sacrificed with excess pentobarbital sodium, the tumors were removed and weighed.

### Immunohistochemical

Briefly, the tumor specimens from three groups of mice were fixed in a 4% formaldehyde solution, dehydrated, transparent, and embedded in paraffin for further assay. Paraffin-embedded tissue was cut into 4 μm-thick sections. The sections were first dewaxed, hydrated, and antigenically repaired. Then, the sections were incubated in 3% H_2_O_2_ for 15 min at 37°C to inhibit endogenous peroxidase. Next, the sections were blocked using 10% goat serum at room temperature for 30 min, then incubated with the primary antibody overnight at 4°C. Primary antibodies included rabbit anti-caspase-3 (1:100, CST, United States), mouse anti-STAT3 (1: 500, CST, United States), and mouse anti-caspase-9 (1: 100, Abcam, United States). On the second day, the sections were incubated with the secondary antibody for 30 min, and then incubated with DAB and hematoxylin. Finally, tissue sections were photographed for analysis.

### Statistical analysis

The data were presented as the means ± standard deviations (SD). The Student’s t-test was used to analyze the differences between the two groups. Comparison among three or more groups was conducted using a one-way analysis of variance (ANOVA) followed by Tukey test. A two-side *p*-value <0.05 was considered statistically significant. All *in vitro* experiments were repeated three times.

## Results

### Extraction and structural formula of compounds

**FIGURE 1:**
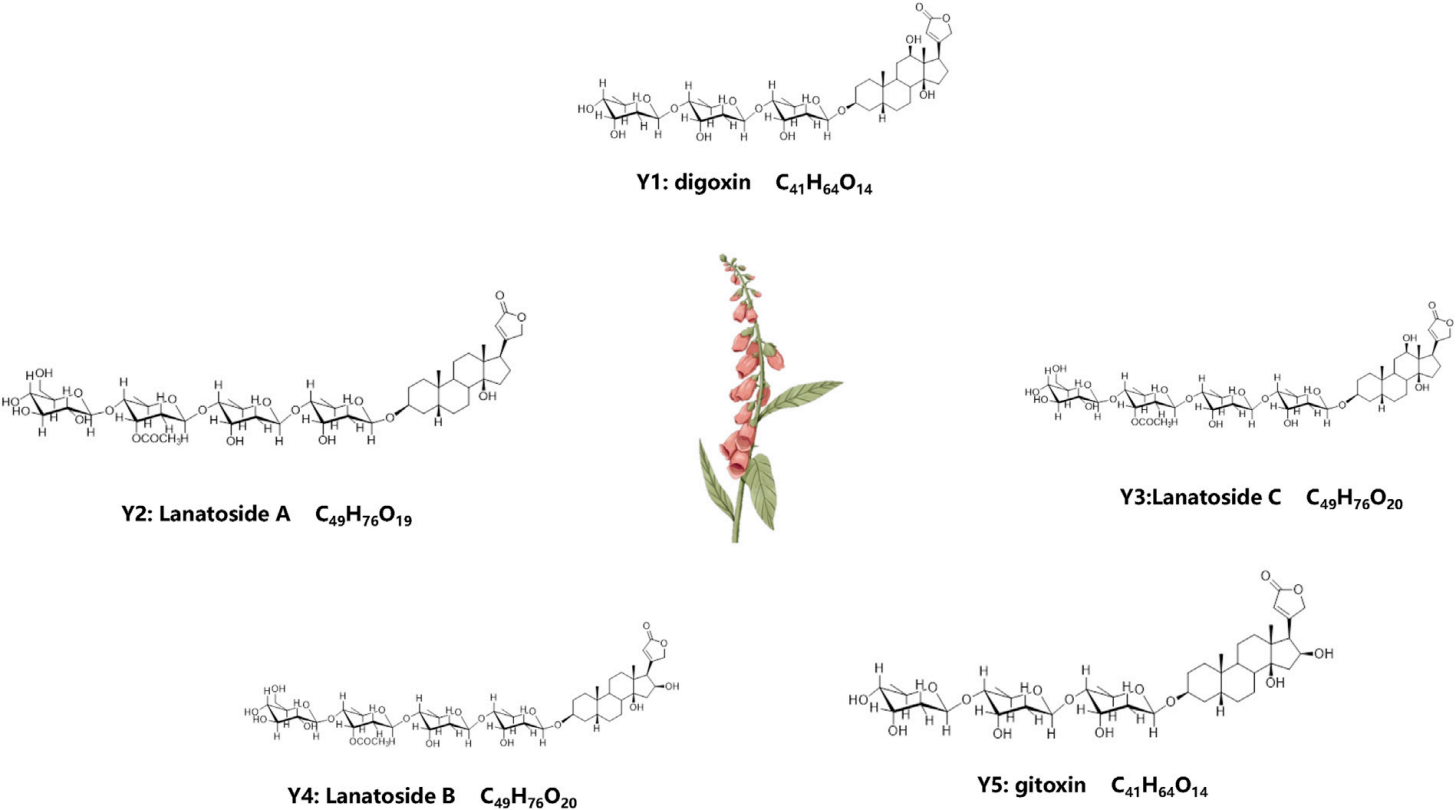
Molecular formula and chemical structure of 5 compounds extracted from plant D. lanata. Y1: Digoxin, Y2: Lanatoside A, Y3: Lanatoside C, Y4: Lanatoside B, Y5: Gitoxin.

After isolation and purification, we isolated five compounds from *D. lanata*: Y1-Y5, and were identified as digoxin (Y1), lanatoside A (Y2), lanatoside C (Y3), lanatoside B (Y4) and gitoxin (Y5), (Figure 1). Their structures were elucidated by comparing their NMR spectral data with the literature (Lehtola et al., 1981; Ren et al., 2020) (Supplementary Figure S1, Supplementary Table S1). The IC50 value of Lan C in HIBEpiC was significantly higher than that in the two bile duct cancer cells, suggesting that Lan C is safe (Supplementary Figure S2).

### Lan C inhibits the proliferation of cholangiocarcinoma cells and has strong anticancer activity

**FIGURE 2:**
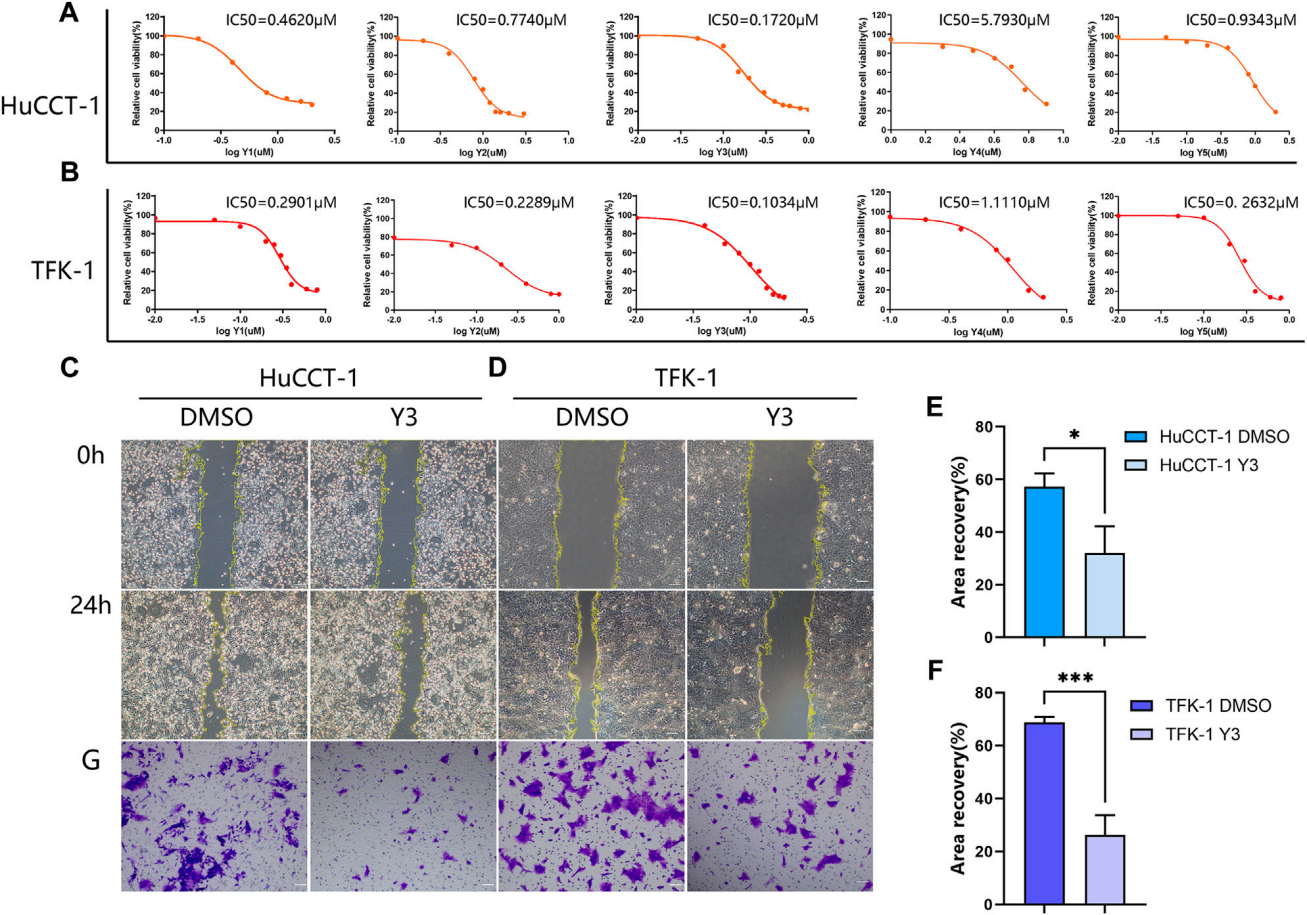
Lan C inhibited the proliferation of CCA. **(A, B)** The IC_50_ values of five drugs (Y1-Y5) in HuCCT-1 and TFK-1 cell lines. **(C, E)** Representative images of wound healing after 24 h of Y3 treatment in HuCCT-1 cell, the therapeutic concentration of Y3 was 0.1720 µM. **(D, F)** Representative images of wound healing after 24 h of Y3 treatment in TFK-1 cell, the therapeutic concentration of Y3 was 0.1034uM. **(G)** Representative images of transwell assay after 24 h of Y3 treatment in HuCCT-1 and TFK-1 cell. *Represents *p*-value less than 0.05, ***Represents *p*-value less than 0.001. Scale bars, 100 μm.

The results of the CCK-8 assay showed that five compounds were cytotoxic action to the HuCCT-1 and TFK-1 (Figures 2A, B). Based on the results of IC_50_, Lan C was finally selected as an ideal compound for subsequent studies. Wound healing and transwell assay confirmed that the viability of HuCCT1 and TFK-1 cells decreased significantly after Lan C treatment (Figures 2C–G). However, the cytotoxic effect on HIBEpiC cell was less (Supplementary Figure S3). The live cell imaging assay showed that the increase of cholangiocarcinoma cells in the experimental group was significantly inhibited compared with the control group. Representative images of HuCCT-1 and TFK-1 cells at 0, 12, 24, and 48 h were acquired in Cytation 5 (Supplementary Figures S4A–D). Our work reveals that Lan C has strong anti-cholangiocarcinoma activity.

### Lanatoside C induced cholangiocarcinoma cell cycle arrest and apoptosis

**FIGURE 3:**
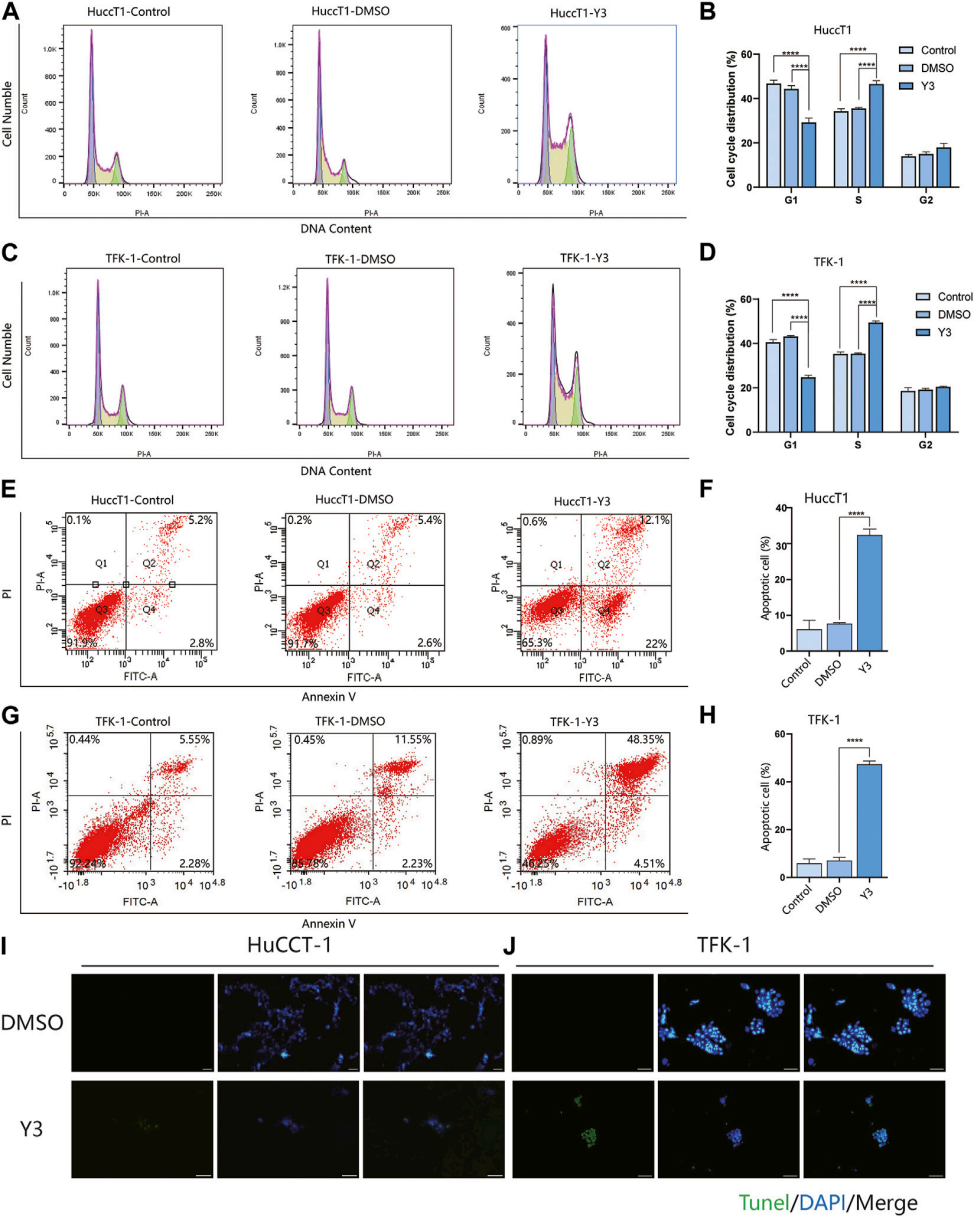
Y3 suppresses cell growth and induces apoptosis in CCA cells. **(A, B)** HuCCT-1 cells were treated with Y3 for 48h, and cell cycle distribution was determined by flow cytometry. **(C, D)** TFK-1 cells were treated with Y3 for 48h, and cell cycle distribution was determined by flow cytometry. **(E, F)** The HuCCT-1 cell line was treated with Y3 for 48h, percentage of cell apoptosis was determined by Annexin-V/PI staining and flow cytometry. **(G, H)** The TFK-1 cell line was treated with Y3 for 48h, percentage of cell apoptosis was determined by Annexin-V/PI staining and flow cytometry. **(I, J)** HuCCT-1 and TFK-1 cells were treated with Y3 for 48h, and the changes in apoptosis were detected by TUNEL assay. In the figure, the therapeutic concentration of Y3 for HuCCT-1 cell line was 0.1720uM, and that for TFK-1 cell line was 0.1034uM. ****Represents *p*-value less than 0.0001. Scale bars, 20 μm.

Given that cell proliferation depends on cell cycle progression, we therefore investigated the cell cycle phase distribution in Lan C treated HuCCT-1 and TFK-1 cells. Significantly increase in S/G2 phase proportion was observed in cells treated with Lan C compared with control group (Figures 3A–C). Quantitative results showed that Lan C blocked HuCCT-1 and TFK-1 cell cycle progression in S/G2 phase (Figures 3B–D). Usually the anti-proliferation activity was correlated with cell apoptotic response. After 48 h of Lan C treatment, increase in the proportion of apoptosis was observed in both HuCCT-1 and TFK-1 cells (Figures 3E–G). This was also confirmed by quantitative results (Figures 3F–H). Interestingly, these results were significantly inhibited when cells were pretreated with 5 mM NAC for 2 h before exposure to Lan C, confirming ROS involvement in the pro-apoptotic effect of Lan C (Supplementary Figure S5). In addition, we also found that the pro-apoptotic effect of Lan C on HIBEpiC was significantly lower than that on cholangiocarcinoma cells (Supplementary Figure S6). To further demonstrate the promoting effect of Lan C on apoptosis of HuCCT-1 and TFK-1 cells, we performed TUNEL experiments to reveal the apoptosis-inducing effect of Lan C (Figures 3I, J). Taken together, these results suggest that Lan C exhibits significant anticancer activity by inhibiting proliferation and inducing apoptosis of cholangiocarcinoma cells, but has no significant toxic effect on HIBEpiC.

### Transcriptomics and network pharmacology analysis revealed that lan C targets STAT3

**FIGURE 4:**
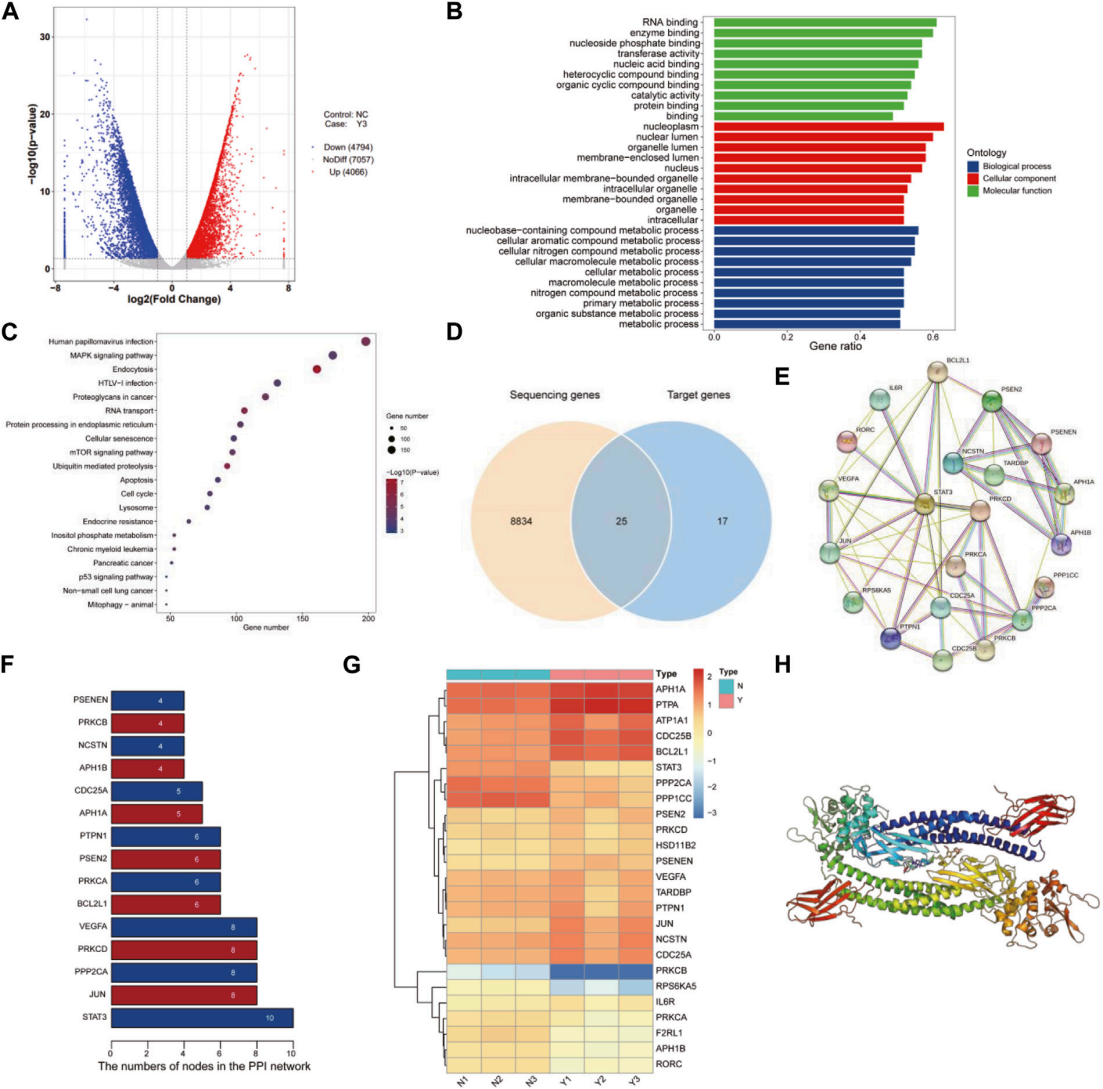
Transcriptomic and network pharmacology analyses. **(A)** Volcano plot of differentially expressed genes. **(B)** GO enrichment analysis of differentially expressed genes. **(C)** KEGG enrichment analysis of differentially expressed genes. **(D)** Venn diagram of differentially expressed genes and compound target genes. **(E)** Protein interaction network analysis of intersecting genes. **(F)** Protein interaction network node degree statistics. **(G)** Heat map of intersection genes. The colors of the tiles in the heat map represent the measured expression value of genes, a three color scale is used with steelblue indicating low expression values, yellow indicating intermediately expressed genes, and brown representing highly expressed genes. **(H)** Compound Lanatoside C and STAT3 molecular docking.

**FIGURE 5:**
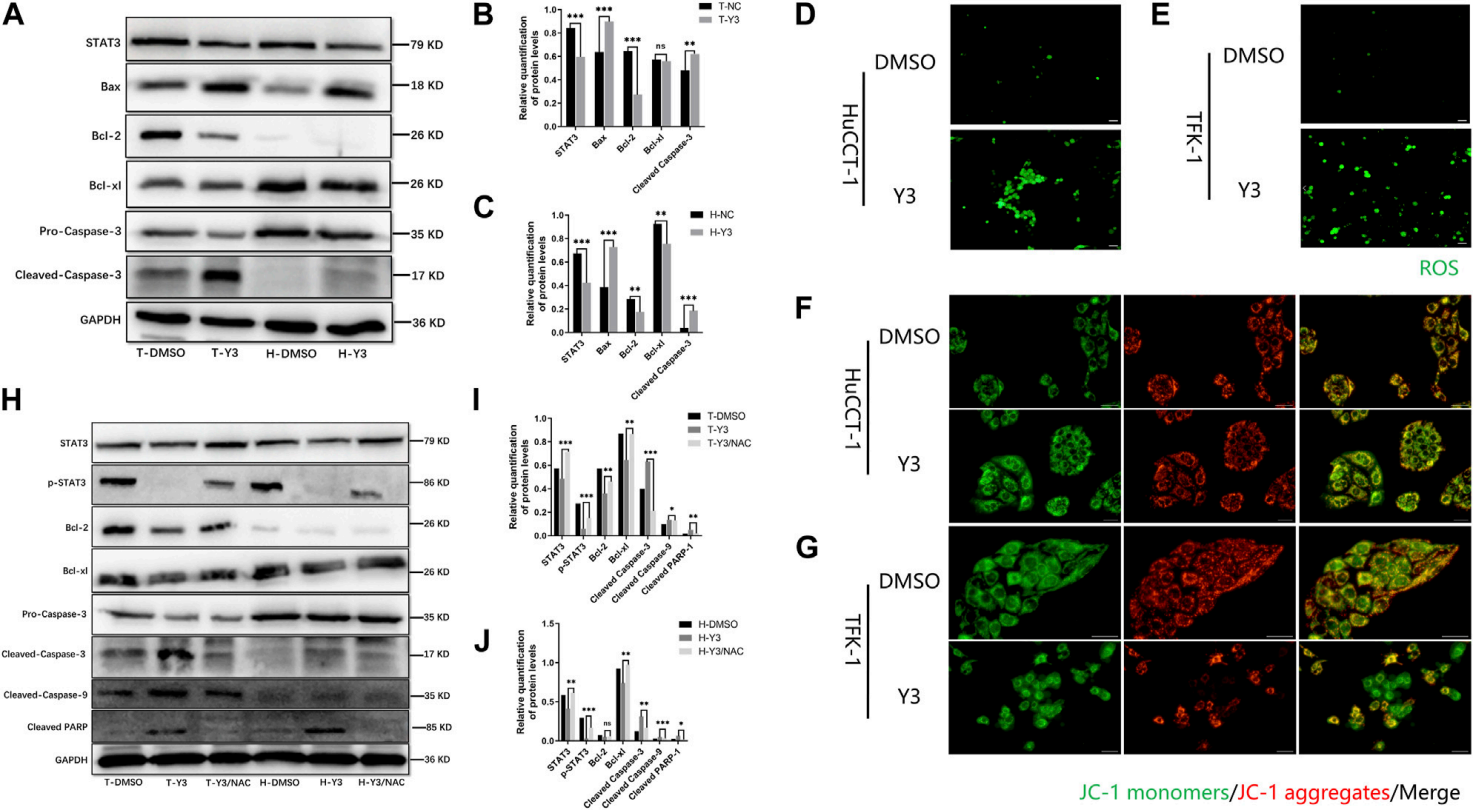
Y3 induces apoptosis by inhibiting STAT3 and ROS-mediated mitochondrial membrane potential transformation. WB and immunofluorescence results. **(A–C)** Western blotting analysis of the expression of STAT3 and apoptotic protein Caspase-3, Bax, Bcl-2 and Bcl-xl in HuCCT-1 and TFK-1 cells after 48 h treatment with Y3. GAPDH served as the loading control. **(D, E)** Fluorescence imaging was used to evaluate ROS generation in HuCCT-1 cell and TFK-1 cell after 48 h treatment with Y3. **(F, G)** HuCCT-1 and TFK-1 cells treated with Y3 were stained using JC-1 dye and examined using fluorescence microscopic analysis, green represents JC-1 monomers, red represents JC-1 aggregates, yellow represents merge images. **(H–J)** Western blotting analysis of the expression of STAT3, p-STAT3 and apoptosis related protein in HuCCT-1 cell and TFK-1 cell after treatment with Y3 or Y3+NAC. In the figure, the therapeutic concentration of Y3 for HuCCT-1 cell line was 0.1720uM, and that for TFK-1 cell line was 0.1034uM. *Represents *p*-value less than 0.05, **Represents *p*-value less than 0.01, ***Represents *p*-value less than 0.001, ns represent *p*-value greater than 0.05. Scale bars, 20 μm.

Transcriptomics and network pharmacology analysis revealed that Lan C targeted STAT3. Utilizing high-throughput transcriptomics sequencing, a total of 8,859 differentially expressed genes (DEGs) were obtained by volcano plot (Figure 4A). We performed functional enrichment analysis to explore the pathways associated with DEGs. The results showed that DEGs were mainly enriched to 2735 GO terms (top 10 of GO terms shown in Figure 4B) and 90 KEGG pathways (top 20 shown in Figure 4C). We obtained 42 target genes of Lan C utilized Comparative Toxicogenomics Database (https://ctdbase.org/) and SwissTargetPrediction (http://www.swisstargetprediction.ch/) database. A Venn diagram showing the intersection of the 25 genes from the two datasets (Figure 4D). We used the STRING database (https://cn.string-db.org/) to explore the protein-protein interaction (Figure 4E) and the top 15 ranked proteins were presented (Figure 4F). We also plotted heatmap of those genes using ggplot2 package (Figure 4G). STAT3 was obtained by PPI network analysis and verified further. Finally, we performed compound Lanatoside C and STAT3 molecular docking, the binding affinity for target protein was −10.1 (Figure 4H). These results were strongly demonstrated in subsequent Western blotting analysis. Lan C inhibited STAT3 expression, which favored the downregulation of Bcl-2 and Bcl-xl and increased the expression of Bax. Meanwhile, the decrease in mitochondrial membrane potential activated caspase-9 and caspase-3. Ultimately, Lan C promoted apoptosis in cholangiocarcinoma cells (Figures 5A–C).

### Lan C induces oxidative stress in cholangiocarcinoma cells

**FIGURE 6:**
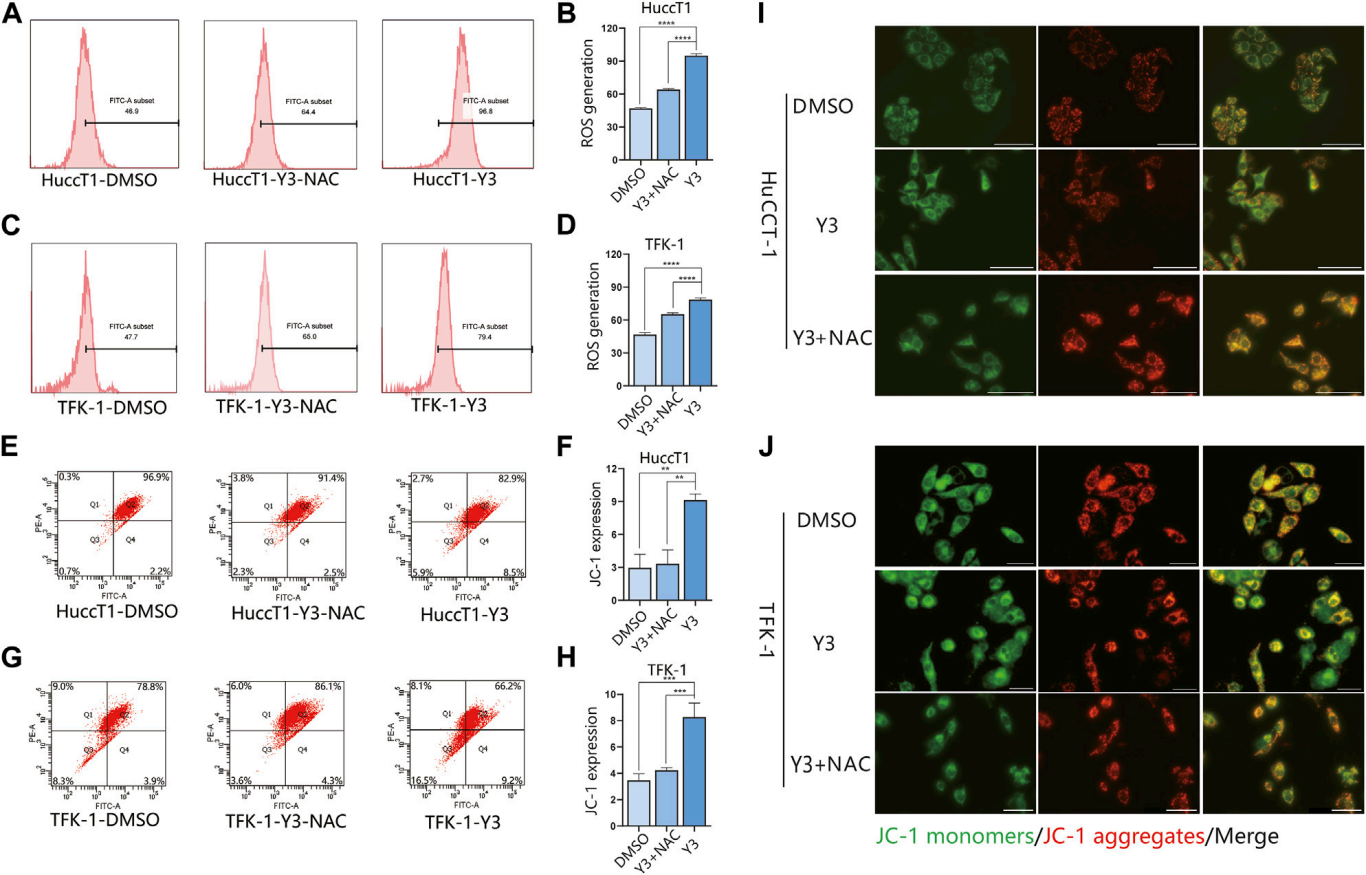
Y3 induces apoptosis by inhibiting STAT3 and ROS-mediated mitochondrial membrane potential transformation. Flow cytometry and immunofluorescence results. **(A, B)** HuCCT-1 cell lines were treated with Y3 or Y3+ NAC, and the changes of ROS were determined by flow cytometry. **(C, D)** TFK-1 cell lines were treated with Y3 or Y3+NAC, and the changes of ROS were determined by flow cytometry **(E, F)** HuCCT-1 cells were treated with Y3 or Y3+NAC, Annexin V/PI staining and flow cytometry were used to detect the changes of JC-1. **(G, H)** TFK-1 cells were treated with Y3 or Y3+NAC, Annexin V/PI staining and flow cytometry were used to detect the changes of JC-1. **(I, J)** HuCCT-1 and TFK-1 cells treated with Y3 or Y3+NAC were stained with JC-1 dye, green represents JC-1 monomers, red represents JC-1 aggregates, yellow represents merged images. In the figure, the therapeutic concentration of Y3 for HuCCT-1 cell line was 0.1720uM, and that for TFK-1 cell line was 0.1034uM. **Represents *p*-value less than 0.01. ***Represents *p*-value less than 0.001. ****Represents *p*-value less than 0.0001. Scale bars, 20 μm.

Current evidence suggests that elevated intracellular reactive oxygen species (ROS) disrupt the integrity of mitochondria, resulting in an intrinsic apoptotic caspase cascade (Rizwan et al., 2020). ROS can act as second messengers, amplifying extracellular signals and delivering them to mitochondria (Yan and Zhao, 2020). Accordingly, we measured ROS levels in cholangiocarcinoma cells treated with Lan C by immunofluorescence. The experimental results confirmed that Lan C treatment resulted in increased ROS levels in HuCCT-1 and TFK-1 cells (Figures 5D, E). NAC is an inhibitor of ROS and can effectively inhibit the effect of ROS (Saxena et al., 2019). This was confirmed by flow cytometry, pretreatment of cells with NAC significantly reversed Lan C induced increase in ROS levels. Removal of ROS significantly attenuated Lan C induced growth inhibition of HuCCT-1 and TFK-1 cells (Figures 6A–D). These results suggest that ROS generation is a key regulator of Lan C induced apoptosis in cholangiocarcinoma cells.

### Changes in mitochondrial membrane potential (MMP) are involved in Lan C induced apoptosis in cholangiocarcinoma cells

We analyzed the effect of MMP in CCA cells treated with Lan C by detecting the changes of JC-I in CCA cells. Immunofluorescence analysis showed that JC-1 was present in the mitochondria of HuCCT-1 and TFK-1 cells as a polymer with bright red fluorescence. In contrast, CCA cells treated with Lan C exhibited decreased mitochondrial membrane potential, and JC-1 was not present in the mitochondrial matrix as a polymer. Green fluorescence in the cytoplasm was significantly enhanced, substantiating that the mitochondrial membrane potential was involved in the apoptosis of CCA cells (Figures 5F, G). Flow cytometry and their quantitative data also confirmed that the amount of JC-1 monomers was significantly increased in HuCCT-1 and TFK-1 cells treated with Lan C. Interestingly, NAC pretreated cells significantly reversed this outcome (Figures 6E–H).

### Lan C induced mitochondrial membrane potential is dependent on ROS production in cholangiocarcinoma cells

Our previous studies have demonstrated that Lan C induces apoptosis in CCA cells leading to an increase in intracellular ROS and a decrease in MMP. Nevertheless, it remains unclear whether there is a relationship between ROS and MMP levels. Current evidence suggests that the decrease in mitochondrial membrane potential may be related to intracellular electron leakage resulting in ROS generatio (Odagiri et al., 2009). In a subsequent experiment, we investigated the effect of the ROS inhibitor NAC on Lan C induced apoptosis in cholangiocarcinoma cells by Western blotting. After NAC pretreatment, STAT3 expression was not downregulated in cholangiocarcinoma cells, the expression of p-STAT3 was significantly increased compared with Y3 group, and caspase-3, caspase-9, cleaved PARP expression were significantly inhibited (Figures 5H–J). The same results were confirmed by immunofluorescence (Figures 6I, J). These results suggest that ROS production may be an upstream regulator of Lan C induced changes in mitochondrial membrane potential, ultimately leading to apoptosis in CCA cells.

### Lan C inhibits the growth of HuCCT-1 xenograft tumors *in Vivo*

**FIGURE 7:**
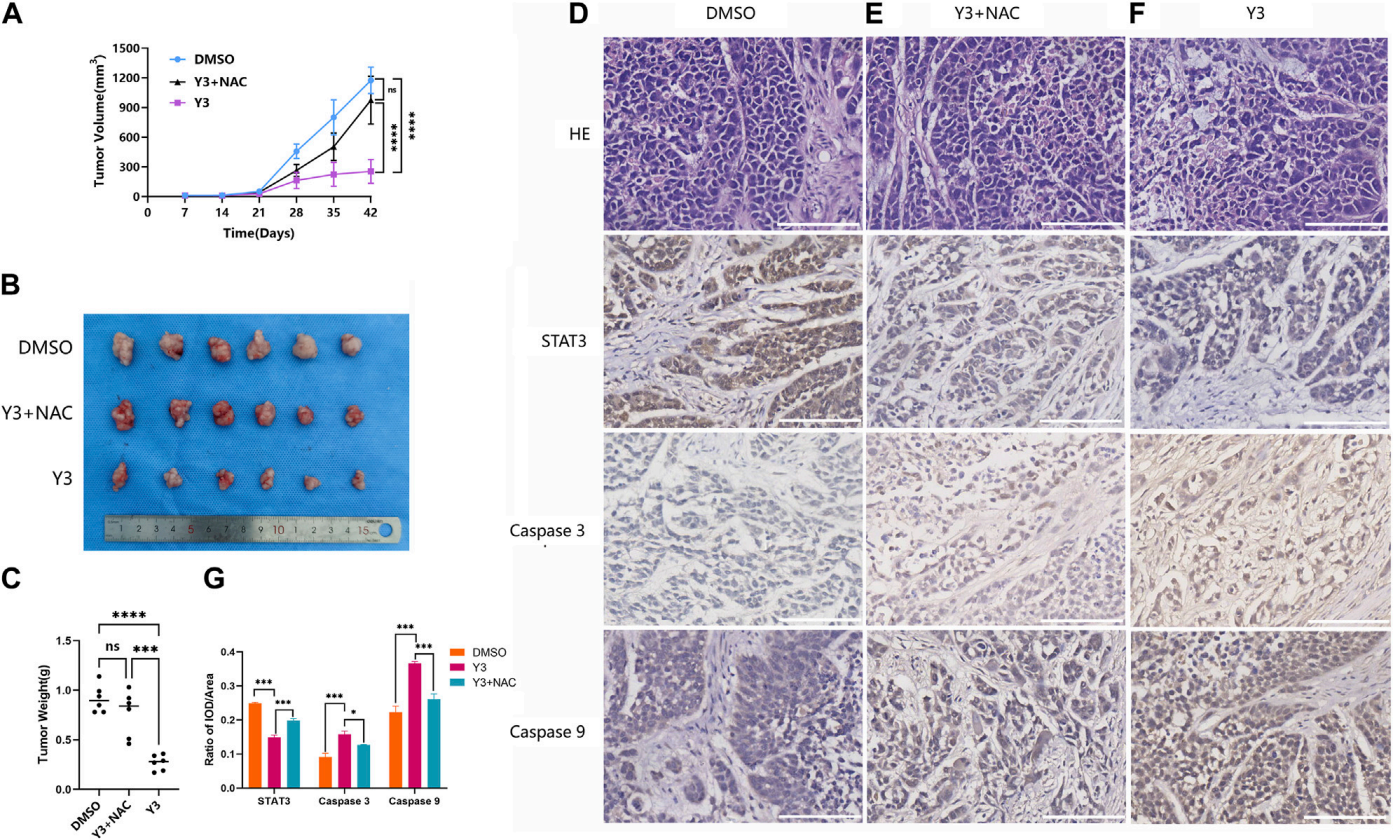
Y3 inhibited CCA progression *in vivo*. **(A)** Tumor volume in three groups of xenografted mice. Circle represents mice treated with 100ul PBS/d, square represents mice treated with Y3 40 mg/kg/d and volume of 100ul, the triangle represents mice treated with NAC + Y3, and 100 mg/kg/d NAC was given by gavage 2 h before Y3 **(B)** Xenograft tumors in the three groups. **(C)** The weight of the three groups of xenograft tumors. **(D–F)** HE staining and immunohistochemical images of STAT3, Caspase-3 and Caspase-9 in the three groups. **(G)** Immunohistochemical statistical results of STAT3, Caspase-3 and Caspase-9 in three groups. *Represents *p*-value less than 0.05, ***Represents *p*-value less than 0.001, ****Represents *p*-value less than 0.0001, ns represent *p*-value greater than 0.05. Scale bars, 100 μm.

To investigate the effect of Lan C on tumor growth *in vivo*, we used a subcutaneously transplanted HuCCT-1 cell model from immunodeficient mice and performed subsequent experiments using gavage. A 40 mg/kg dose of Lan C significantly reduced the volume and weight of HuCCT-1 tumors compared to controls. Importantly, 42 days of Lan C treatment was well tolerated and did not result in significant weight loss (Figures 7A–C; Supplementary Figure S7). The size of the tumor in nude mice given NAC + Lan C intragastric administration was between the control group and the experimental group, indicating that NAC also inhibited the effect of ROS *in vivo*. Furthermore, we assessed the expression levels of major proteins associated with apoptosis in tumors using immunohistochemical assays. We found that Lan C induced a significant increase in caspase-9 and caspase-3 activity and a significant decrease in STAT3 expression in tumor tissues, consistent with the results *in vitro* experiments (Figures 7D–F).

### Lan C inhibits STAT3 expression and promotes apoptosis in cholangiocarcinoma cells mediated by ROS

**FIGURE 8:**
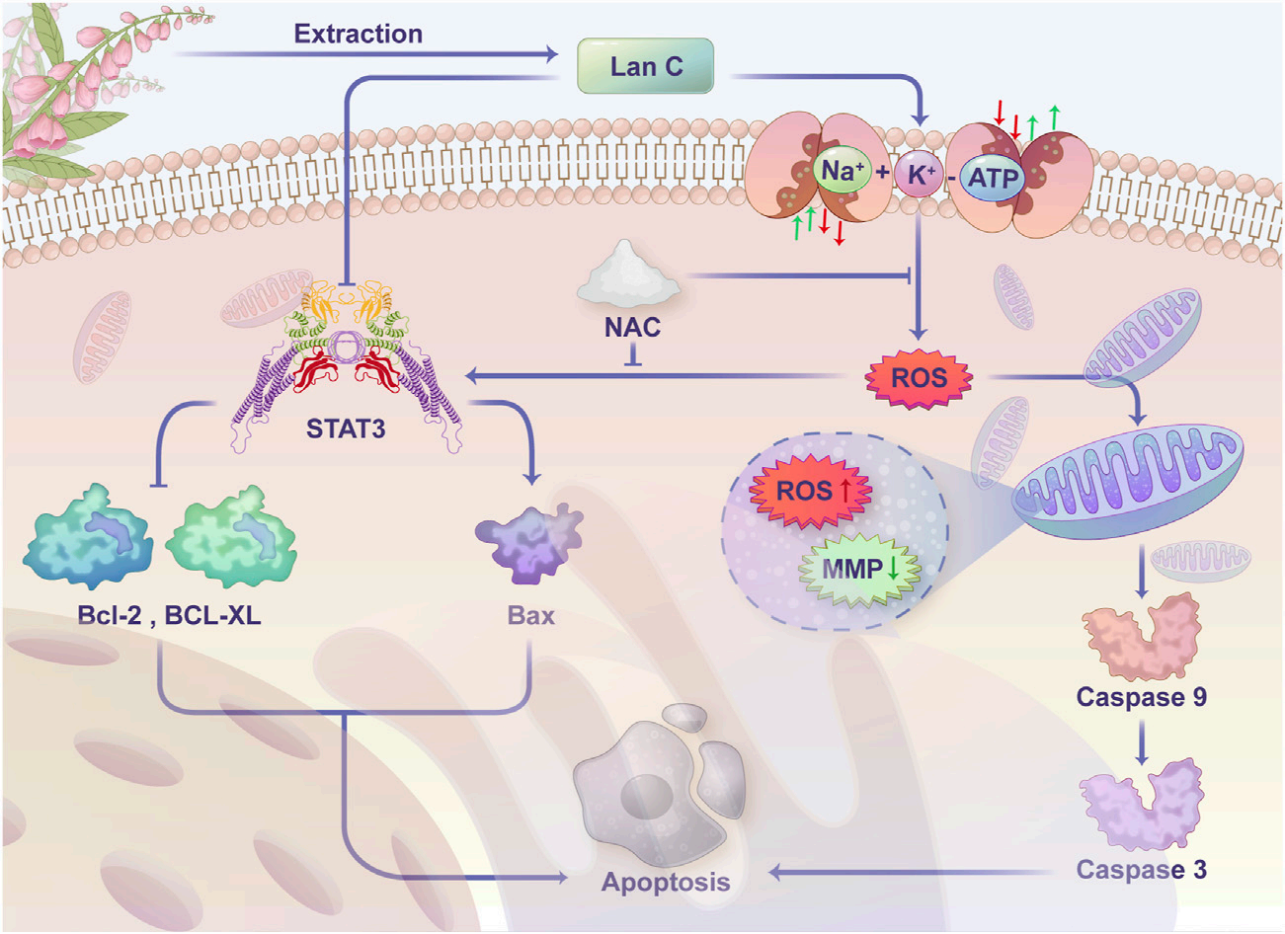
Schematic representation of the proposed anti-cancer effect of Lanatoside C on CCA cells.

We treated HuCCT-1 and TFK-1 cells with Lan C and then detected the expression of STAT3 by Western blot. We found that STAT3 expression was significantly reduced in HuCCT-1 and TFK-1 cells after Lan C treatment. In the present study, we observed that downregulation of STAT3 expression resulted in decreased Bcl-2 and Bcl-xl expression, increased Bax expression, activation of caspase-9, cleaved PARP and caspase-3, and the apoptotic cascade was initiated in HuCCT-1 and TFK-1 cells. In addition, pretreatment of HuCCT-1 and TFK-1 cells by NAC reversed the effect of Lan C on STAT3 and downstream apoptosis-related proteins. These results suggest that Lan C may promote apoptosis of cholangiocarcinoma cells by increasing ROS content while inducing a decrease in STAT3 expression (Figure 8). Taken together, these results suggest that Lan C can not only activate caspase-9 and caspase-3 by inducing an increase in ROS, leading to a decrease in mitochondrial membrane potential, but also inhibit STAT3 expression, resulting in a downregulation of Bcl-2 and Bcl-xl expression and an increase in Bax expression, thus promoting apoptosis in cholangiocarcinoma cells. The above results were also confirmed *in vivo*.

## Discussion

Cardiac glycosides comprise a large family of naturally derived compounds, such as Lan C, digoxin, digitalis toxin and ouabain, which share a common structural parent. Their core structure consists of a steroidal structure, widely thought to be responsible for the pharmacodynamic activity of these compounds (Whayne, 2018). Lan C and digoxin are closely related structurally, as digoxin can be obtained from the hydrolysis of the acetyl and glucose fractions from Lan C. The main pharmacological effect of cardiac glycosides, which are still widely used in clinical practice, is to inhibit cell membrane Na^+^/K^+^-ATPase and indirectly enhance myocardial contractility (Hood et al., 2014; Alpert, 2021). Recent studies have shown that several cardiac glycosides exert anticancer activity through different mechanisms, such as the SRC/EGFR/RAS/ERK signaling pathway, p21, NF-κB, AP-1, topoisomerase, and HIF-1 (Biggar et al., 2011; Biggar, 2012; Liu et al., 2020). These results suggest that cardiac glycosides has huge prospects for clinical application as an anticancer drug. In the present study, we explored the antitumor activity of Lan C in CCA and confirmed that Lan C has strong pro-apoptotic activity mediated by two pathways. On the one hand, increased intracellular ROS can induce a decrease in mitochondrial membrane potential, activating the mitochondrial apoptotic pathway. On the other hand, increased ROS inhibits the pro-oncogenic effect of STAT3, which leads to a decrease in Bcl-2 and Bcl-cl levels and an increase in Bax expression, ultimately causing apoptosis in CCA. Taken together, our findings suggest that ROS is an important target for treatment.

The sodium pump is the target of cardiac glycosides, which have long been used in the treatment of heart failure (Katz, 1985). Recent studies have found that sodium pump expression is higher in colon, prostate, pancreatic, lung and breast cancers. Regardless of the tissue origin, the higher the level of sodium pump expression, the more sensitive the cells were to prednisolone treatment (Bagrov et al., 2002; Silva et al., 2021; Cheung and Vousden, 2022). In the present study, Lan C yielded a strong pro-apoptotic effect on cholangiocarcinoma cells, which strongly suggests the high expression of sodium pump is elevated in cholangiocarcinoma cells. It has been reported that cardiac glycosides can induce Src activation and increase ROS production by reducing Na^+^/K^+^-ATPase activity (Xie and Cai, 2003). Moreover, excess ROS can disrupt the antioxidant defense of cells, alter the mitochondrial genome, increase protein oxidation, and lead to mitochondrial damage (Durmaz et al., 2016). Our study confirmed that Lan C could elevate ROS in cholangiocarcinoma cells, thus causing mitochondrial dysfunction. Interestingly, ROS may act as a second messenger to transmit extracellular signals inside the cells during this process.

Redox reactions are related to energy metabolism redox and are indispensable for life itself. It is widely acknowledged that ROS generation and elimination systems actively maintain the intracellular redox state, mediate redox signaling and regulate cellular functions (Rigoulet et al., 2011; Shadel and Horvath, 2015). Current evidence suggests that reactive oxygen species play a subtle role in regulating various aspects of cellular function, targeting and modifying the function, localization and activity of widely distributed proteins in a strictly regulated and reversible manner. For example, activating a large number of cell surface receptors increases intracellular hydrogen peroxide levels, thereby mediating downstream effects in a second messenger-like manner (Shi et al., 2017; Moloney and Cotter, 2018; Srinivas et al., 2019).

Mitochondria represent the main site of ROS production and the target of ROS (Zorov et al., 2014). Under specific pathological conditions, the accumulation of excessive ROS can disrupt intracellular homeostasis, leading to oxidative stress and mitochondrial dysfunction, which can damage the mitochondrial lipid membrane, leading to alterations in the mitochondrial membrane potential and apoptosis (Wang et al., 2018). In the present study, the action of Lan C on cholangiocarcinoma cells increased intracellular ROS production and inhibited the pro-oncogenic function of the transcription factor STAT3. ROS acts as the target molecule for the anti-cancer effect of Lan C. When the intracellular content of ROS increases, Bax protein may be recruited to the mitochondrial surface to form pore channels and promote the apoptosis of cholangiocarcinoma cells. In addition, ROS levels in mitochondria increased rapidly, resulting in a decrease in mitochondrial membrane potential, which activated Caspase-9, cleaved PARP and Caspase-3 and promoted apoptosis in CCA. This phenomenon was reversed by NAC, an inhibitor of ROS, reversing the switching effect of ROS.

Mitochondrial function is a key indicator of cellular health and can be assessed by monitoring changes in mitochondrial membrane potential (MMP) (Demine et al., 2019). Mitochondrial dysfunction has been associated with various diseases, such as cancer, cardiovascular disease, diabetes and neurodegenerative diseases (Bock and Tait, 2020). Many compounds can reduce MMP by interfering with multiple macromolecules in mitochondria, affecting mitochondrial function (Battogtokh et al., 2018). JC-1 has been extensively used to evaluate MMP over the years. In the present study, we used JC-1 staining to detect changes in mitochondrial membrane potential in cholangiocarcinoma cells after treated with Lan C. After cholangiocarcinoma cells were treated with Lan C action, a change from red to green fluorescence was observed under fluorescence staining. The ratio of JC-1 polymer/JC-1 monomer was significantly decreased, indicating that Lan C could cause membrane depolarization, resulting in decreased MMP. Flow cytometry also confirmed that Lan C caused loss of mitochondrial membrane potential in CCA cells, which resulted in apoptosis. Blockade of ROS production by NAC completely reversed Lan C-induced changes in mitochondrial membrane potential and apoptosis, suggesting that the Lan C-induced decrease in mitochondrial membrane potential and apoptosis were dependent on ROS production.

The signal transducer and activator of transcription (STAT) is a family of transcription factors, of which STAT3 is associated with signaling from cell surface receptors to the nucleus and has been a research hot in recent years (Haghikia et al., 2014; Zou et al., 2020). Various cancers, including cholangiocarcinoma, frequently exhibit STAT3 activation (Fathi et al., 2018). Accordingly, compounds that can dysregulate STAT3 activation have huge therapeutic potential, and blocking the STAT3 signaling pathway may lead to cancer cell growth inhibition and apoptosis. In our study, sequencing and network pharmacology analysis suggested that STAT3 represents a poteneially important target of Lan C action on cholangiocarcinoma cells, confirmed in subsequent WB and *in vivo* experiments. Lan C caused a decrease in the protein expression of STAT3 in cholangiocarcinoma cells, which inhibited the classical STAT3 pro-cancer pathway and led to apoptosis of cancer cells. In the same experimental group, the change of p-STAT3 was consistent with that of STAT3, suggesting that Lan C may play a pro-apoptotic role by inhibiting the phosphorylation of STAT3. In contrast, NAC pretreatment reversed this change, suggesting that this effect is also associated with ROS. Thus, our study reveals a unique approach to target STAT3 via ROS-induced anticancer compounds, providing a promising avenue for treating cholangiocarcinoma.

Herein, we aimed to elucidate the molecular mechanism underlying the anticancer effect of Lan C in cholangiocarcinoma. We found that Lan C caused an increase in intracellular ROS content, which in turn caused a decrease in the mitochondrial membrane potential of cholangiocarcinoma cells and eventually led to apoptosis. Lan C also downregulated the protein expression of STAT3 through ROS, increased the expression of Bax, decreased Bcl-2 and Bcl-xl levels, and activated caspase-9, cleaved PARP and caspase-3, which initiated apoptosis. These results suggest that Lan C may be a new anti-cholangiocarcinoma compound. However, our study has some limitations. First, the molecular mechanism of lanc’s antitumor effect needs further investigation. Besides, little is currently known about the interactions between ROS and STAT3. Based on the strong oxidative properties of ROS, we speculate that this characteristic may be critical in affecting STAT3 function. Indeed, further research is warranted before clinical translation.

## Conclusion

In conclusion, our results provide hitherto undocumented evidence of the mechanism of action of Lan C in cholangiocarcinoma. We demonstrated that Lan C inhibits the growth of cholangiocarcinoma cells and promotes their apoptosis by increasing ROS production, decreasing mitochondrial membrane potential and inhibiting the pro-oncogenic effect of STAT3. Overall our results suggest that Lan C could be acandidate compounds for cholangiocarcinoma.

## Data Availability

The datasets presented in this study can be found in online repositories. The names of the repository/repositories and accession number(s) can be found in the article/Supplementary Material.

## Supplementary material

SUPPLEMENTARY FIGURE S1 NMR spectrum of compounds Y1-Y5.

SUPPLEMENTARY FIGURE S2 The IC50 value of Y3 in human intrahepatic bile duct epithelial cell line (HIBEpiC) is 0.2124 µM/L.

SUPPLEMENTARY FIGURE S3 Cytotoxic effects of Lan C on HuCCT-1 cells and HIBEpiC cells.

SUPPLEMENTARY FIGURE S4 Live cell imaging assay. **(A, B)** The cell number of HuCCT-1 and TFK-1 cells treated with Y3 for 48 h were monitored using Cytation 5. **(C, D)** Representative images acquired in Cytation 5 of HuCCT-1 and TFK-1 cells at 0, 12, 24, and 48 h.

SUPPLEMENTARY FIGURE S5 The HuCCT-1 and TFK-1 cells line were treated with Y3 or NAC + Y3 for 48 h, percentage of cell apoptosis was determined by Annexin-V/PI staining and flow cytometry.

SUPPLEMENTARY FIGURE S6 The HIBEpiC cell line was treated with Y3 or NAC + Y3 for 48 h, percentage of cell apoptosis was determined by Annexin-V/PI staining and flow cytometry.

SUPPLEMENTARY FIGURE S7 Weight of tumor xenografts mice treated with PBS, Y3 and NAC + Y3.

## References

[B1] Alpert J. S. (2021). Is digitalis therapy still viable? Foxglove therapy makes a comeback. Am. J. Med. 134, 1–2. 10.1016/j.amjmed.2020.09.001 32980337

[B2] Anders S. Huber W. J. E. (2013). Differential expression of RNA-Seq data at the gene level – the DESeq package. Germany: EMBL.

[B3] Bagrov A. Y. Bagrov Y. Y. Fedorova O. V. Kashkin V. A. Patkina N. A. Zvartau E. E. (2002). Endogenous digitalis-like ligands of the sodium pump: Possible involvement in mood control and ethanol addiction. Eur. Neuropsychopharmacol. J. Eur. Coll. Neuropsychopharmacol. 12, 1–12. 10.1016/s0924-977x(01)00127-4 11788235

[B4] Battogtokh G. Choi Y. S. Kang D. S. Park S. J. Shim M. S. Huh K. M. (2018). Mitochondria-targeting drug conjugates for cytotoxic, anti-oxidizing and sensing purposes: Current strategies and future perspectives. Acta Pharm. Sin. B 8, 862–880. 10.1016/j.apsb.2018.05.006 30505656 PMC6251809

[B5] Biggar R. J. (2012). Molecular pathways: Digoxin use and estrogen-sensitive cancers--risks and possible therapeutic implications. Clin. Cancer Res. Official J. Am. Assoc. Cancer Res. 18, 2133–2137. 10.1158/1078-0432.CCR-11-1389 22368159

[B6] Biggar R. J. Wohlfahrt J. Oudin A. Hjuler T. Melbye M. (2011). Digoxin use and the risk of breast cancer in women. J. Clin. Oncol. Official J. Am. Soc. Clin. Oncol. 29, 2165–2170. 10.1200/JCO.2010.32.8146 21422417

[B7] Bock F. J. Tait S. W. G. (2020). Mitochondria as multifaceted regulators of cell death. Nat. Rev. Mol. Cell Biol. 21, 85–100. 10.1038/s41580-019-0173-8 31636403

[B8] Chang J. Li Y. Wang X. Hu S. Wang H. Shi Q. (2017). Polyphyllin I suppresses human osteosarcoma growth by inactivation of Wnt/β-catenin pathway *in vitro* and *in vivo* . Sci. Rep. 7, 7605. 10.1038/s41598-017-07194-9 28790389 PMC5548759

[B9] Chen G. Zhou D. Li X.-Z. Jiang Z. Tan C. Wei X.-Y. (2017). A natural chalcone induces apoptosis in lung cancer cells: 3D-QSAR, docking and an *in vivo*/vitro assay. Sci. Rep. 7, 10729. 10.1038/s41598-017-11369-9 28878321 PMC5587747

[B10] Chen S. Zhou Y. Chen Y. Gu J. (2018). fastp: an ultra-fast all-in-one FASTQ preprocessor. Bioinformatics 34, i884–i890. 10.1093/bioinformatics/bty560 30423086 PMC6129281

[B11] Cheung E. C. Vousden K. H. (2022). The role of ROS in tumour development and progression. Nat. Rev. Cancer 22, 280–297. 10.1038/s41568-021-00435-0 35102280

[B12] Demine S. Renard P. Arnould T. (2019). Mitochondrial uncoupling: A key controller of biological processes in physiology and diseases. Cells 8, 795. 10.3390/cells8080795 31366145 PMC6721602

[B13] Durmaz I. Guven E. B. Ersahin T. Ozturk M. Calis I. Cetin-Atalay R. (2016). Liver cancer cells are sensitive to Lanatoside C induced cell death independent of their PTEN status. Phytomedicine Int. J. Phytotherapy Phytopharm. 23, 42–51. 10.1016/j.phymed.2015.11.012 26902406

[B14] Ehle M. Patel C. Giugliano R. P. (2011). Digoxin: Clinical highlights: A review of digoxin and its use in contemporary medicine. Crit. Pathw. Cardiol. 10, 93–98. 10.1097/HPC.0b013e318221e7dd 21988950

[B15] Fathi N. Rashidi G. Khodadadi A. Shahi S. Sharifi S. (2018). STAT3 and apoptosis challenges in cancer. Int. J. Biol. Macromol. 117, 993–1001. 10.1016/j.ijbiomac.2018.05.121 29782972

[B16] Haghikia A. Ricke-Hoch M. Stapel B. Gorst I. Hilfiker-Kleiner D. (2014). STAT3, a key regulator of cell-to-cell communication in the heart. Cardiovasc. Res. 102, 281–289. 10.1093/cvr/cvu034 24518140

[B17] Hood W. B. Dans A. L. Guyatt G. H. Jaeschke R. Mcmurray J. J. V. (2014). Digitalis for treatment of heart failure in patients in sinus rhythm. Cochrane Database Syst. Rev. 2014, CD002901. 10.1002/14651858.CD002901.pub3 24771511 PMC7138042

[B18] Hsu I. L. Chou C.-Y. Wu Y.-Y. Wu J.-E. Liang C.-H. Tsai Y.-T. (2016). Targeting FXYD2 by cardiac glycosides potently blocks tumor growth in ovarian clear cell carcinoma. Oncotarget 7, 62925–62938. 10.18632/oncotarget.7497 26910837 PMC5325337

[B19] Hu Y. Yu K. Wang G. Zhang D. Shi C. Ding Y. (2018). Lanatoside C inhibits cell proliferation and induces apoptosis through attenuating Wnt/β-catenin/c-Myc signaling pathway in human gastric cancer cell. Biochem. Pharmacol. 150, 280–292. 10.1016/j.bcp.2018.02.023 29475060

[B20] Katz A. M. (1985). Effects of digitalis on cell biochemistry: Sodium pump inhibition. J. Am. Coll. Cardiol. 5, 16A–21A. 10.1016/s0735-1097(85)80459-9 2580875

[B21] Kaushik V. Yakisich J. S. Azad N. Kulkarni Y. Venkatadri R. Wright C. (2017). Anti-tumor effects of cardiac glycosides on human lung cancer cells and lung tumorspheres. J. Cell. Physiology 232, 2497–2507. 10.1002/jcp.25611 27662422

[B22] Kelley R. K. Bridgewater J. Gores G. J. Zhu A. X. (2020). Systemic therapies for intrahepatic cholangiocarcinoma. J. Hepatology 72, 353–363. 10.1016/j.jhep.2019.10.009 31954497

[B23] Kim D. Langmead B. Salzberg S. L. (2015). Hisat: A fast spliced aligner with low memory requirements. Nat. Methods 12, 357–360. 10.1038/nmeth.3317 25751142 PMC4655817

[B24] Kreis W. (2017). The foxgloves (digitalis) revisited. Planta Medica 83, 962–976. 10.1055/s-0043-111240 28561136

[B25] Lehtola T. Huhtikangas A. Hiltunen R. V Schantz M. (1981). Radioimmunoassay of digoxigenin glycosides in digitalis lanata. Planta Medica 42, 250–254. 10.1055/s-2007-971635 17401969

[B26] Liu S.-H. Yu J. Creeden J. F. Sutton J. M. Markowiak S. Sanchez R. (2020). Repurposing metformin, simvastatin and digoxin as a combination for targeted therapy for pancreatic ductal adenocarcinoma. Cancer Lett. 491, 97–107. 10.1016/j.canlet.2020.08.002 32829010 PMC8766172

[B27] Mazzaferro V. Gorgen A. Roayaie S. Droz Dit Busset M. Sapisochin G. (2020). Liver resection and transplantation for intrahepatic cholangiocarcinoma. J. Hepatology 72, 364–377. 10.1016/j.jhep.2019.11.020 31954498

[B28] Moloney J. N. Cotter T. G. (2018). ROS signalling in the biology of cancer. Seminars Cell and Dev. Biol. 80, 50–64. 10.1016/j.semcdb.2017.05.023 28587975

[B29] Moriya Y. Itoh M. Okuda S. Yoshizawa A. C. Kanehisa M. (2007). Kaas: An automatic genome annotation and pathway reconstruction server. Nucleic Acids Res. 35, W182–W185. 10.1093/nar/gkm321 17526522 PMC1933193

[B30] Odagiri K. Katoh H. Kawashima H. Tanaka T. Ohtani H. Saotome M. (2009). Local control of mitochondrial membrane potential, permeability transition pore and reactive oxygen species by calcium and calmodulin in rat ventricular myocytes. J. Mol. Cell. Cardiol. 46, 989–997. 10.1016/j.yjmcc.2008.12.022 19318235

[B31] Pratt R. D. Brickman C. R. Cottrill C. L. Shapiro J. I. Liu J. (2018). The Na/K-ATPase signaling: From specific ligands to general reactive oxygen species. Int. J. Mol. Sci. 19, 2600. 10.3390/ijms19092600 30200500 PMC6163532

[B32] Rasheduzzaman M. Yin H. Park S.-Y. (2019). Cardiac glycoside sensitized hepatocellular carcinoma cells to TRAIL via ROS generation, p38MAPK, mitochondrial transition, and autophagy mediation. Mol. Carcinog. 58, 2040–2051. 10.1002/mc.23096 31392779

[B33] Razumilava N. Gores G. J. (2014). Cholangiocarcinoma. Lancet (London, Engl. 383, 2168–2179. 10.1016/S0140-6736(13)61903-0 PMC4069226 24581682

[B34] Reddy D. Kumavath R. Ghosh P. Barh D. (2019). Lanatoside C induces G2/M cell cycle arrest and suppresses cancer cell growth by attenuating MAPK, Wnt, JAK-STAT, and PI3K/AKT/mTOR signaling pathways. Biomolecules 9, 792. 10.3390/biom9120792 31783627 PMC6995510

[B35] Ren Y. Ribas H. T. Heath K. Wu S. Ren J. Shriwas P. (2020). Na+/K+-ATPase-Targeted cytotoxicity of (+)-Digoxin and several semisynthetic derivatives. J. Nat. Prod. 83, 638–648. 10.1021/acs.jnatprod.9b01060 32096998 PMC7243443

[B36] Rigoulet M. Yoboue E. D. Devin A. (2011). Mitochondrial ROS generation and its regulation: Mechanisms involved in H(2)O(2) signaling. Antioxidants Redox Signal. 14, 459–468. 10.1089/ars.2010.3363 20649461

[B37] Rizvi S. Gores G. J. (2013). Pathogenesis, diagnosis, and management of cholangiocarcinoma. Gastroenterology 145, 1215–1229. 10.1053/j.gastro.2013.10.013 24140396 PMC3862291

[B38] Rizwan H. Pal S. Sabnam S. Pal A. (2020). High glucose augments ROS generation regulates mitochondrial dysfunction and apoptosis via stress signalling cascades in keratinocytes. Life Sci. 241, 117148. 10.1016/j.lfs.2019.117148 31830478

[B39] Rodrigues P. M. Olaizola P. Paiva N. A. Olaizola I. Agirre-Lizaso A. Landa A. (2021). Pathogenesis of cholangiocarcinoma. Annu. Rev. Pathology 16, 433–463. 10.1146/annurev-pathol-030220-020455 33264573

[B40] Sato K. Glaser S. Alvaro D. Meng F. Francis H. Alpini G. (2020). Cholangiocarcinoma: Novel therapeutic targets. Expert Opin. Ther. Targets 24, 345–357. 10.1080/14728222.2020.1733528 32077341 PMC7129482

[B41] Saxena S. Vekaria H. Sullivan P. G. Seifert A. W. (2019). Connective tissue fibroblasts from highly regenerative mammals are refractory to ROS-induced cellular senescence. Nat. Commun. 10, 4400. 10.1038/s41467-019-12398-w 31562333 PMC6764955

[B42] Shadel G. S. Horvath T. L. (2015). Mitochondrial ROS signaling in organismal homeostasis. Cell 163, 560–569. 10.1016/j.cell.2015.10.001 26496603 PMC4634671

[B43] Shi X. Li W. Liu H. Yin D. Zhao J. (2017). The ROS/NF-κB/NR4A2 pathway is involved in H2O2 induced apoptosis of resident cardiac stem cells via autophagy. Oncotarget 8, 77634–77648. 10.18632/oncotarget.20747 29100414 PMC5652805

[B44] Silva C. I. D. Gonçalves-De-Albuquerque C. F. Moraes B. P. T. D. Garcia D. G. Burth P. (2021). Na/K-ATPase: Their role in cell adhesion and migration in cancer. Biochimie 185, 1–8. 10.1016/j.biochi.2021.03.002 33713729

[B45] Srinivas U. S. Tan B. W. Q. Vellayappan B. A. Jeyasekharan A. D. (2019). ROS and the DNA damage response in cancer. Redox Biol. 25, 101084. 10.1016/j.redox.2018.101084 30612957 PMC6859528

[B46] Wang Y. Branicky R. Noë A. Hekimi S. (2018). Superoxide dismutases: Dual roles in controlling ROS damage and regulating ROS signaling. J. Cell Biol. 217, 1915–1928. 10.1083/jcb.201708007 29669742 PMC5987716

[B47] Wang Y. Dong B. Xue W. Feng Y. Yang C. Liu P. (2020). Anticancer effect of radix astragali on cholangiocarcinoma *in vitro* and its mechanism via network pharmacology. Med. Sci. Monit. Int. Med. J. Exp. Clin. Res. 26, e921162. 10.12659/MSM.921162 PMC7154565 32246704

[B48] Whayne T. F. (2018). Clinical use of digitalis: A state of the art review. Am. J. Cardiovasc. Drugs Drugs, Devices, Other Interventions 18, 427–440. 10.1007/s40256-018-0292-1 30066080

[B49] Xie C.-M. Liu X.-Y. Yu S. Cheng C. H. K. (2013). Cardiac glycosides block cancer growth through HIF-1α- and NF-κB-mediated Plk1. Carcinogenesis 34, 1870–1880. 10.1093/carcin/bgt136 23615397

[B50] Xie Z. Cai T. (2003). Na+-K+--ATPase-mediated signal transduction: From protein interaction to cellular function. Mol. Interv. 3, 157–168. 10.1124/mi.3.3.157 14993422

[B51] Yan T. Zhao Y. (2020). Acetaldehyde induces phosphorylation of dynamin-related protein 1 and mitochondrial dysfunction via elevating intracellular ROS and Ca2+ levels. Redox Biol. 28, 101381. 10.1016/j.redox.2019.101381 31756635 PMC6879985

[B52] Yan Y. Shapiro J. I. (2016). The physiological and clinical importance of sodium potassium ATPase in cardiovascular diseases. Curr. Opin. Pharmacol. 27, 43–49. 10.1016/j.coph.2016.01.009 26891193 PMC5161351

[B53] Zhang J. Su G. Tang Z. Wang L. Fu W. Zhao S. (2018). Curcumol exerts anticancer effect in cholangiocarcinoma cells via down-regulating CDKL3. Front. Physiology 9, 234. 10.3389/fphys.2018.00234 PMC5870041 29615928

[B54] Zorov D. B. Juhaszova M. Sollott S. J. (2014). Mitochondrial reactive oxygen species (ROS) and ROS-induced ROS release. Physiol. Rev. 94, 909–950. 10.1152/physrev.00026.2013 24987008 PMC4101632

[B55] Zou S. Tong Q. Liu B. Huang W. Tian Y. Fu X. (2020). Targeting STAT3 in cancer immunotherapy. Mol. Cancer 19, 145. 10.1186/s12943-020-01258-7 32972405 PMC7513516

