# Supplementary figures and images for: Lanatoside C decelerates proliferation and induces apoptosis through inhibition of STAT3 and ROS-mediated mitochondrial membrane potential transformation in cholangiocarcinoma
Source: Front Pharmacol. 2023 Jun 15;14:1098915. doi: 10.3389/fphar.2023.1098915 (PMC10308052; doi:10.3389/fphar.2023.1098915)

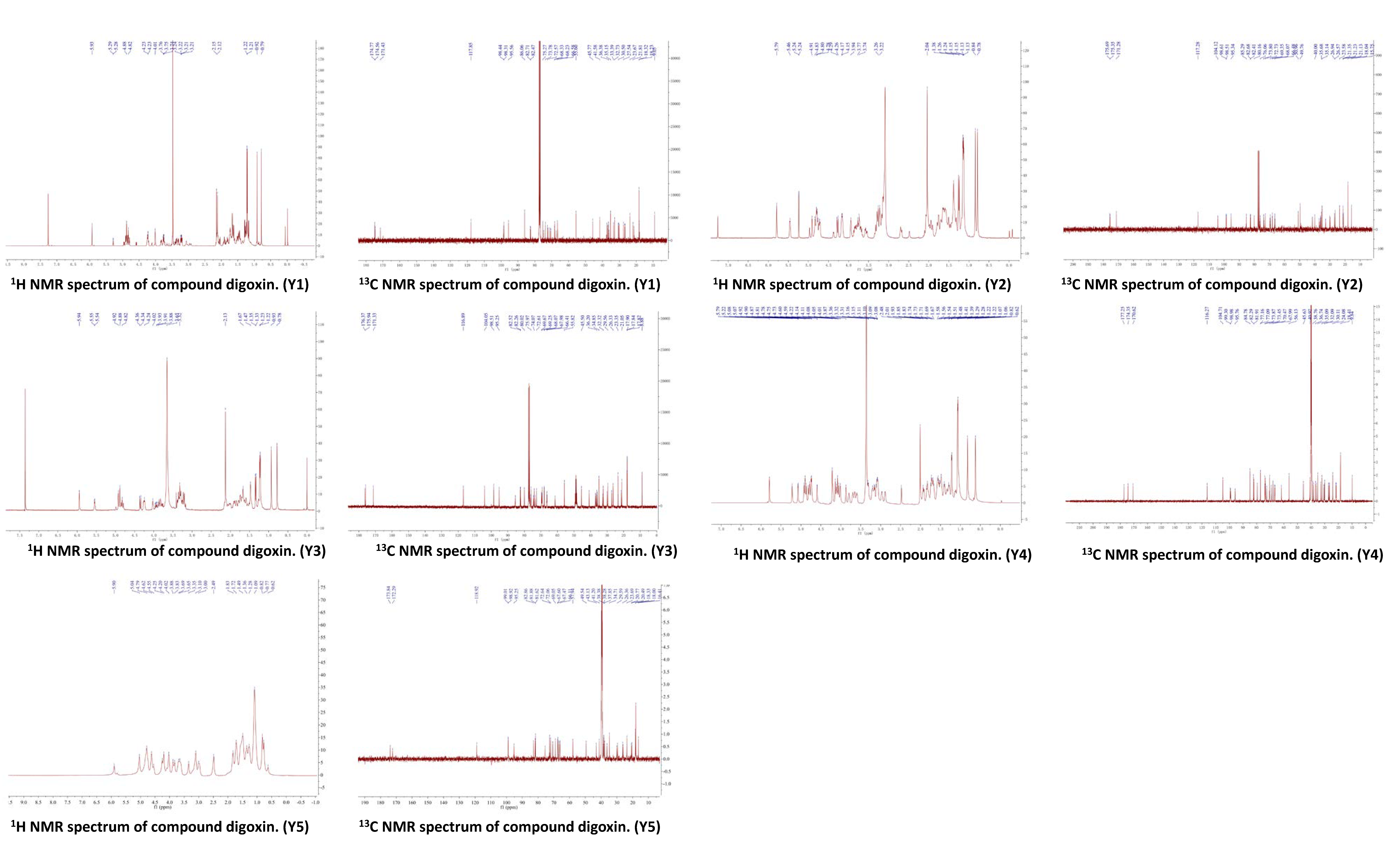

Supplement: Supplementary file 1 [file Image1.tiff]

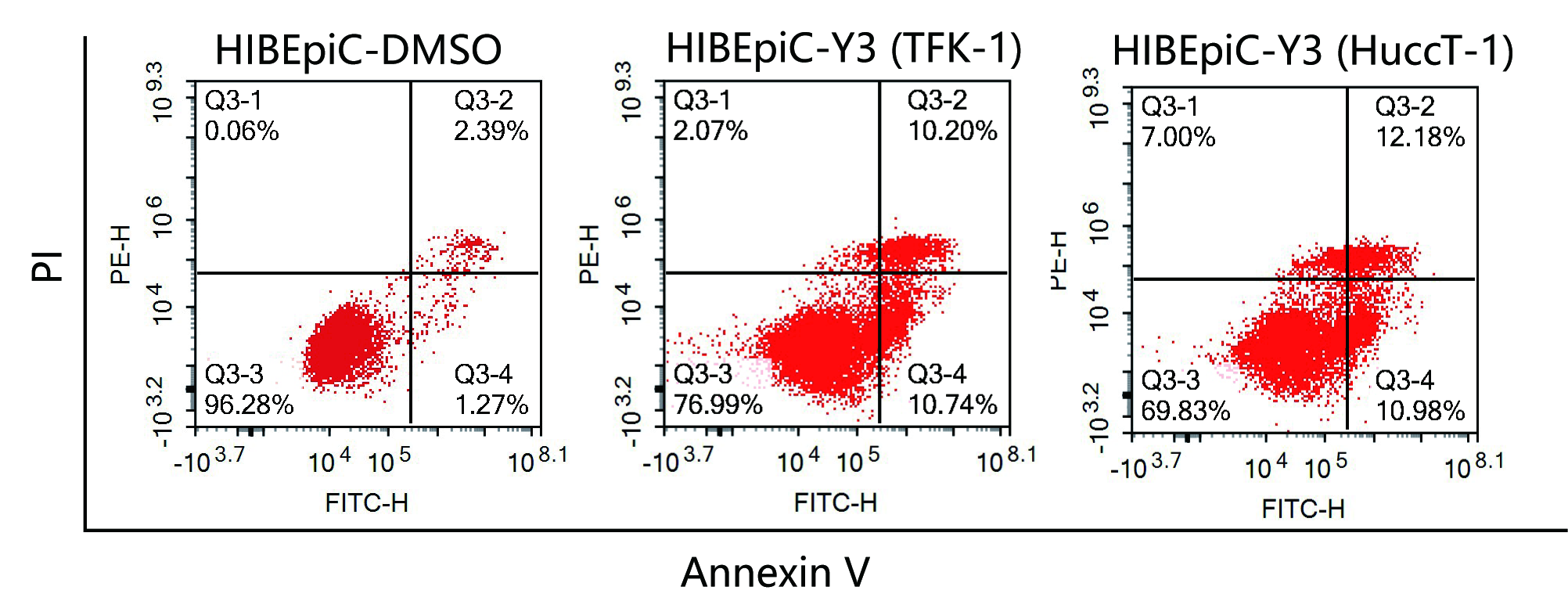

Supplement: Supplementary file 2 [file Image6.tif]

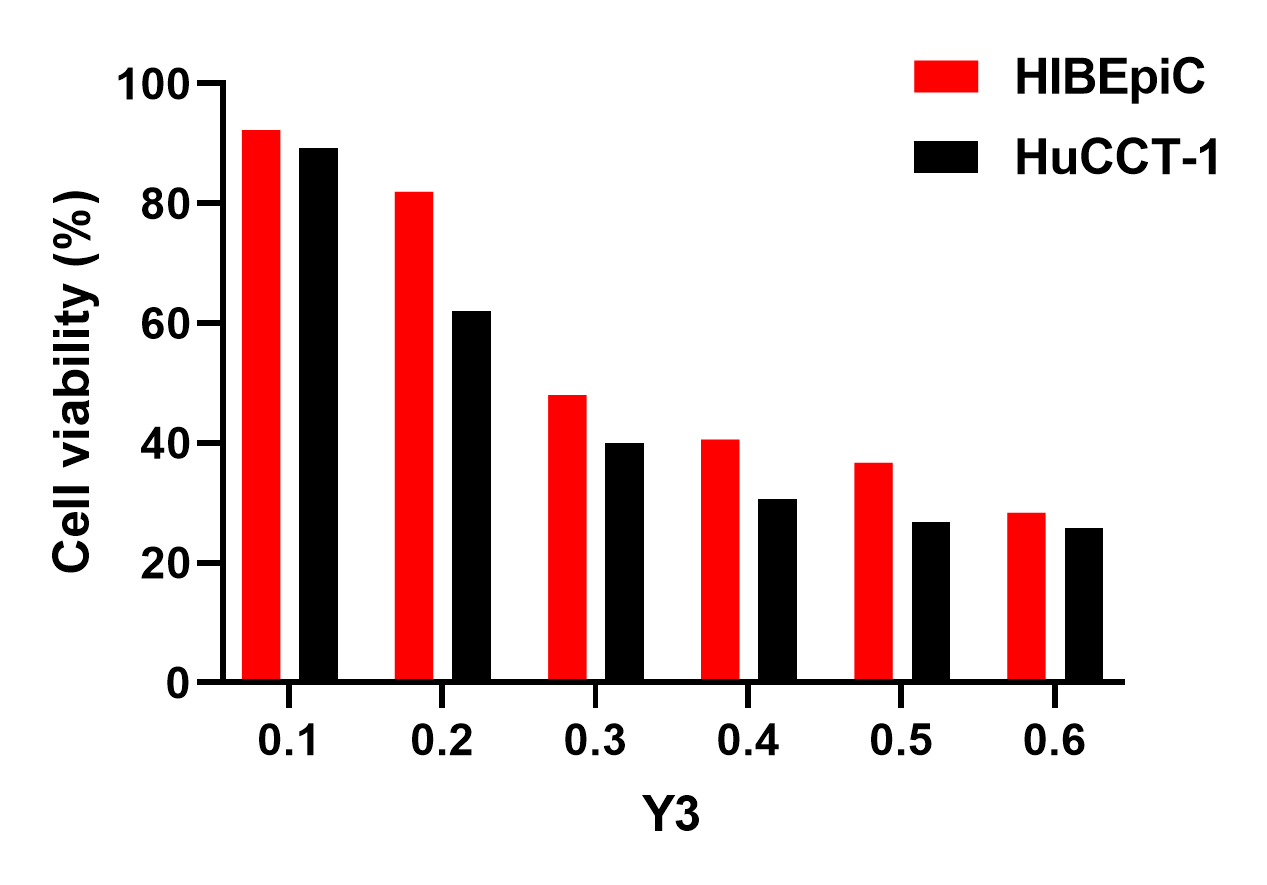

Supplement: Supplementary file 4 [file Image3.tif]

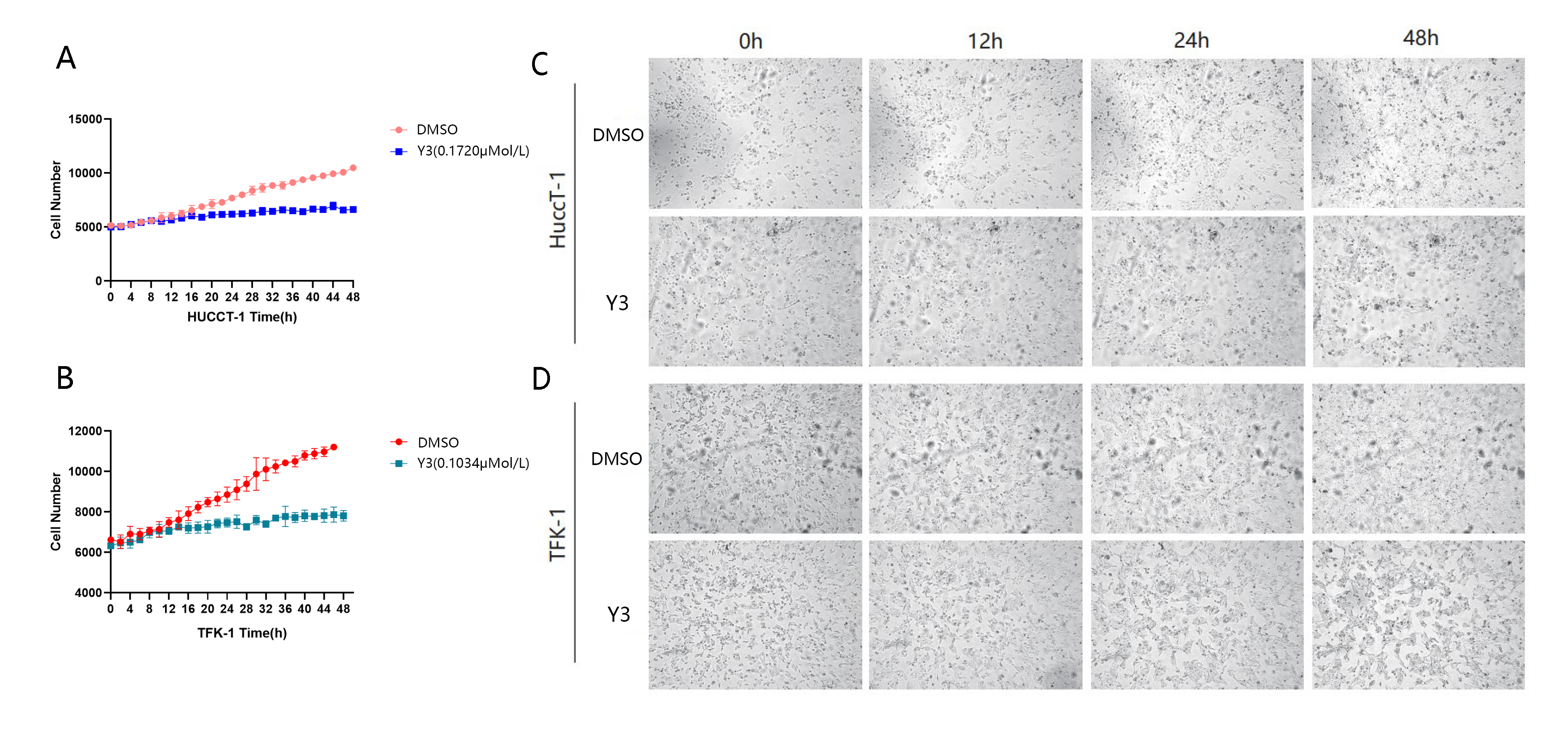

Supplement: Supplementary file 5 [file Image4.tif]

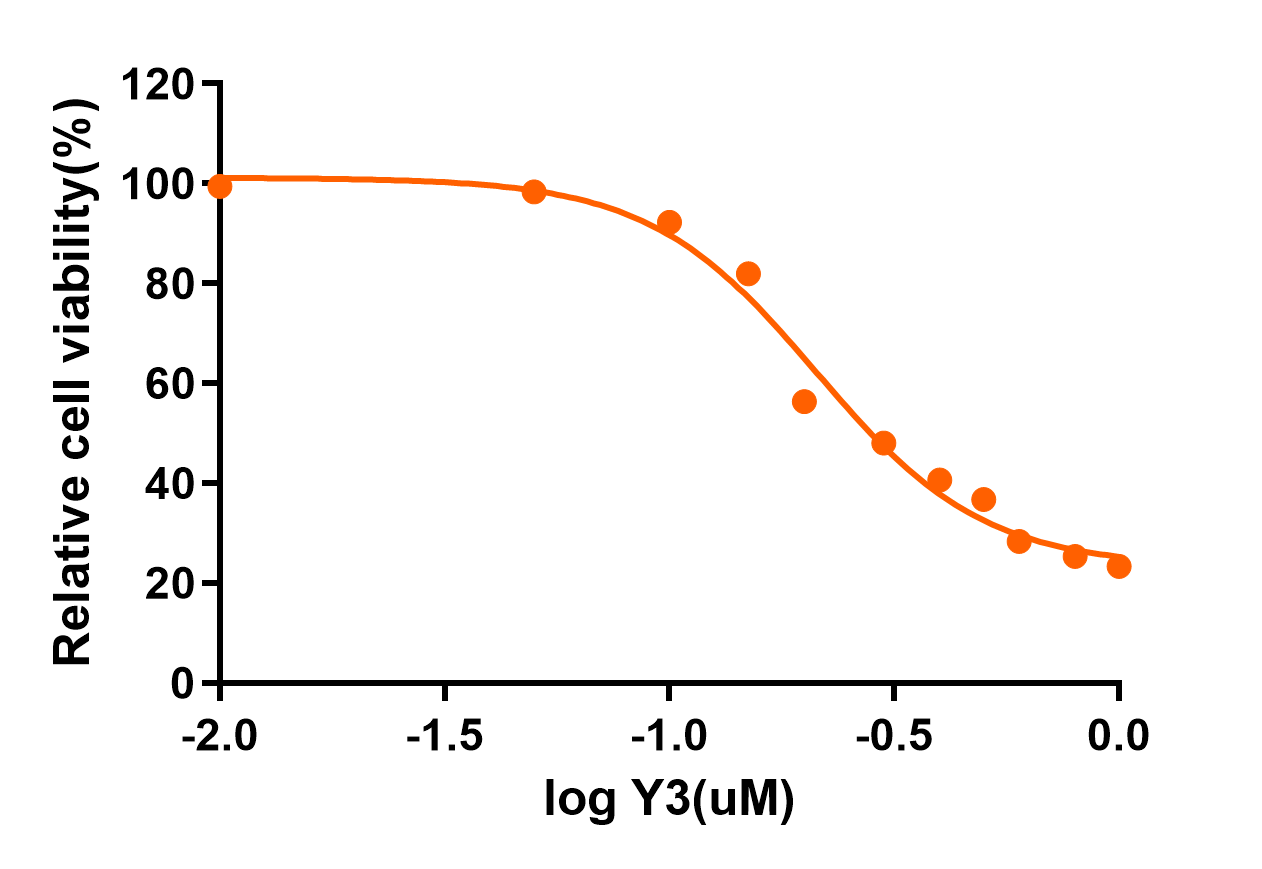

Supplement: Supplementary file 6 [file Image2.tif]

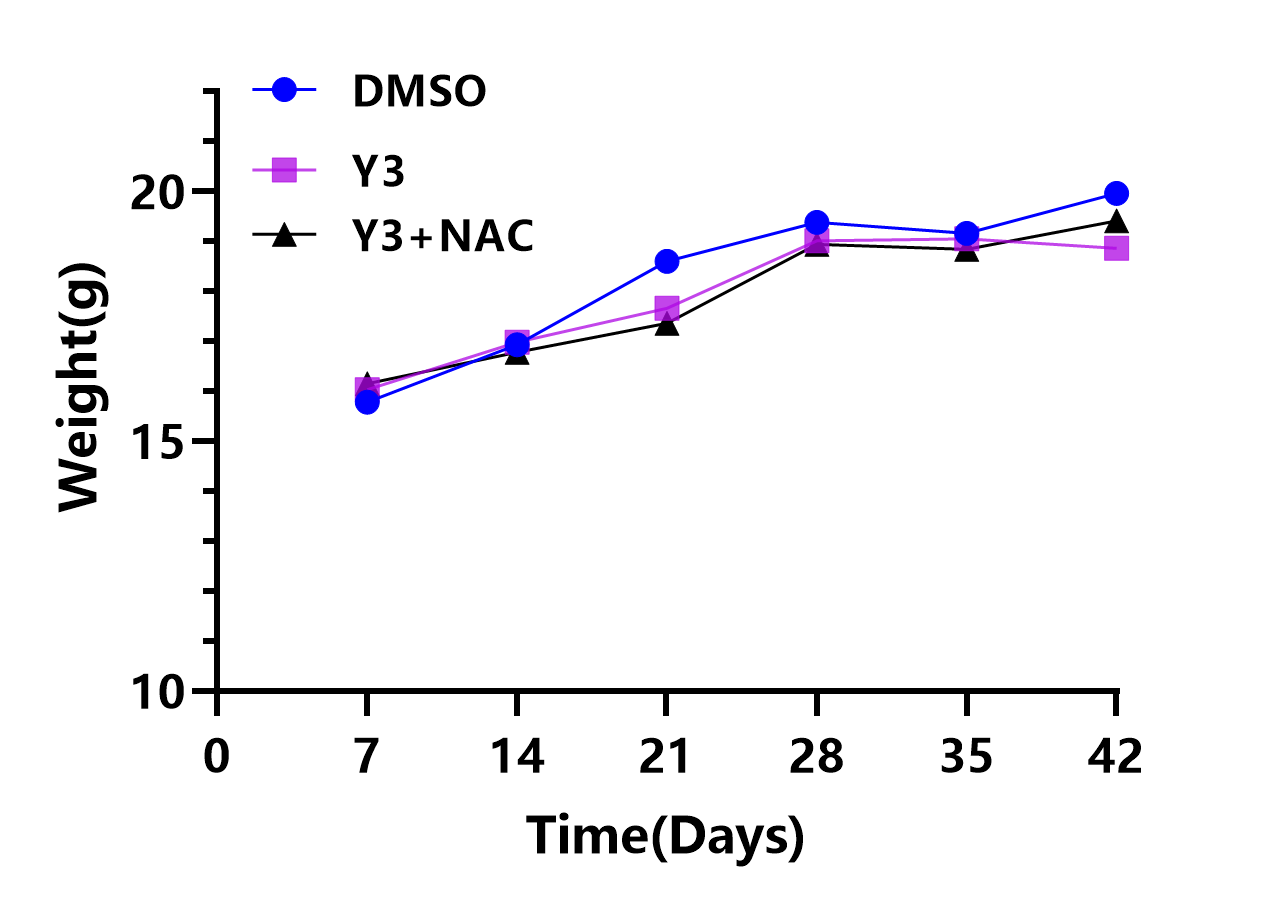

Supplement: Supplementary file 7 [file Image7.tif]

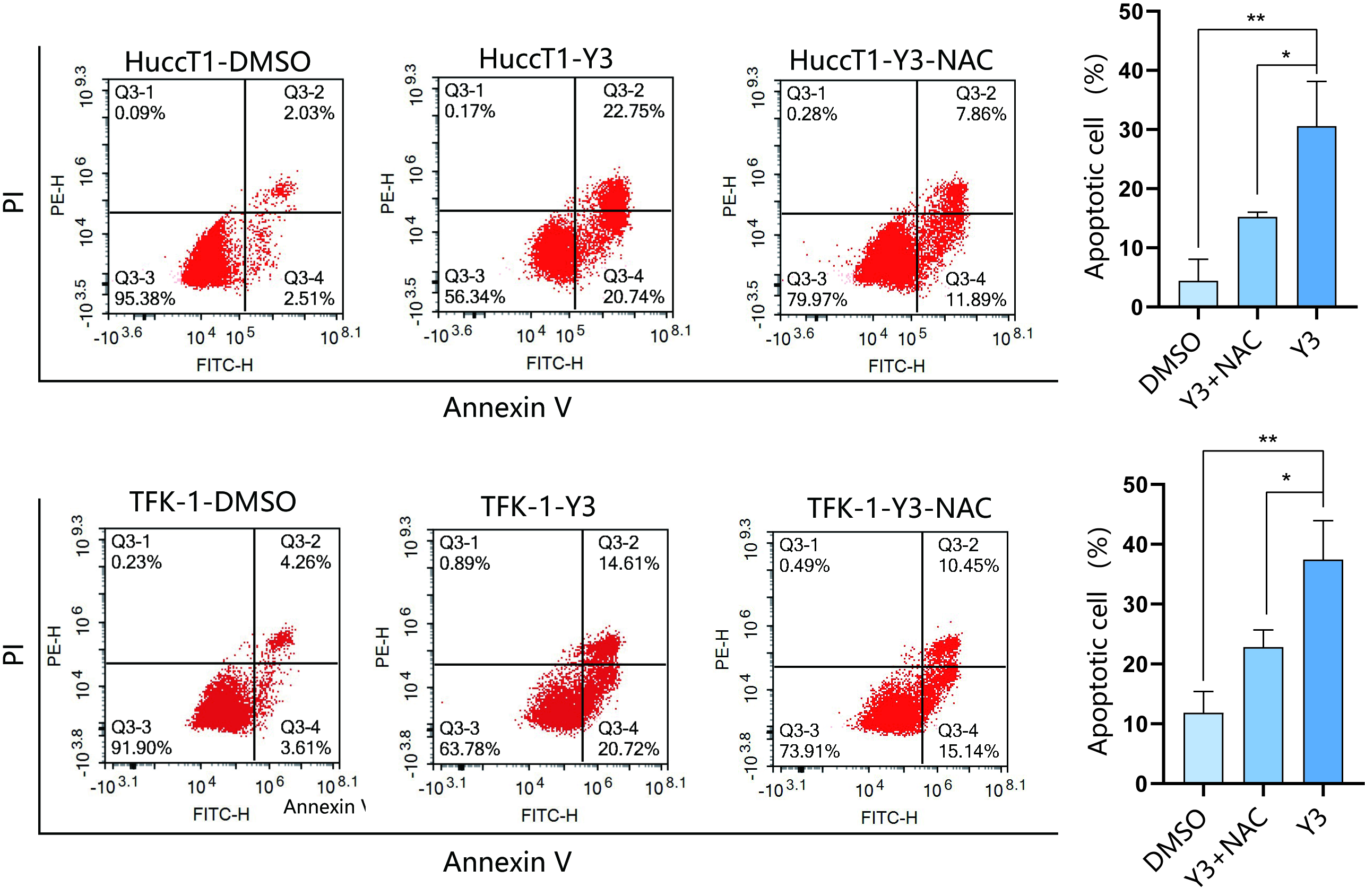

Supplement: Supplementary file 8 [file Image5.tif]
