# Supplementary material for: Lanatoside C decelerates proliferation and induces apoptosis through inhibition of STAT3 and ROS-mediated mitochondrial membrane potential transformation in cholangiocarcinoma
Source: Front Pharmacol. 2023 Jun 15;14:1098915. doi: 10.3389/fphar.2023.1098915 (PMC10308052; doi:10.3389/fphar.2023.1098915)
Supplement: Supplementary file 3 [file Table1.DOCX]

**Table S 1**. ^13^C (150 MHz) NMR data of compounds **1**–**5** (in CDCl_3_).

| No. | *δ* _C_ | | | | |
| --- | --- | --- | --- | --- | --- |
|  | **Y1** | **Y2** | **Y3** | **Y4** | **Y5** |
| 1 | 29.73 | 29.69 | 29.6 | 30.13 | 29.59 |
| 2 | 26.49 | 26.54 | 26.3 | 26.57 | 26.04 |
| 3 | 72.59 | 72.69 | 72.6 | 72.64 | 72.06 |
| 4 | 29.80 | 30.13 | 29.5 | 29.79 | 30.04 |
| 5 | 36.32 | 36.09 | 35.9 | 35.18 | 36.23 |
| 6 | 26.49 | 26.54 | 26.3 | 26.57 | 26.36 |
| 7 | 21.61 | 21.11 | 20.4 | 20.97 | 20.49 |
| 8 | 41.11 | 41.60 | 40.9 | 41.38 | 41.20 |
| 9 | 32.49 | 35.66 | 34.9 | 35.60 | 34.71 |
| 10 | 35.00 | 35.12 | 36.1 | 36.26 | 37.85 |
| 11 | 30.16 | 21.22 | 30.0 | 21.63 | 29.59 |
| 12 | 74.69 | 40.00 | 74.1 | 41.19 | 41.20 |
| 13 | 55.86 | 49.71 | 55.8 | 49.87 | 49..94 |
| 14 | 85.64 | 85.32 | 85.5 | 85.22 | 86.35 |
| 15 | 32.85 | 32.83 | 32.6 | 42.02 | 43.13 |
| 16 | 27.38 | 26.92 | 27.2 | 72.76 | 72.64 |
| 17 | 45.63 | 50.91 | 45.5 | 58.21 | 57.94 |
| 18 | 8.980 | 15.73 | 8.9 | 16.62 | 16.41 |
| 19 | 23.50 | 23.55 | 23.4 | 23.56 | 23.69 |
| 20 | 176.07 | 175.27 | 175.9 | 171.36 | 172.3 |
| 21 | 74.10 | 73.73 | 74.49 | 76.1 | 75.7 |
| 22 | 117.19 | 117.34 | 116.89 | 119.45 | 118.9 |
| 23 | 175.85 | 175.58 | 176.4 | 175.42 | 173.8 |
| 1’ | 95.39 | 95.29 | 95.3 | 95.38 | 95.25 |
| 2’ | 36.77 | 37.06 | 37.0 | 37.13 | 38.28 |
| 3’ | 66.50 | 66.44 | 66.9 | 66.52 | 66.11 |
| 4’ | 82.32 | 82.40 | 82.3 | 82.43 | 81.62 |
| 5’ | 67.73 | 68.05 | 68.1 | 69.38 | 67.47 |
| 6’ | 18.06 | 18.02 | 17.9 | 18.06 | 18.00 |
| 1’’ | 98.58 | 98.43 | 98.5 | 98.53 | 98.92 |
| 2’’ | 37.88 | 36.71 | 36.7 | 36.80 | 38.38 |
| 3’’ | 66.32 | 66.32 | 66.3 | 66.40 | 66.25 |
| 4’’ | 82.66 | 82.65 | 82.5 | 82.67 | 81.88 |
| 5’’ | 68.14 | 68.14 | 68.3 | 68.19 | 67.60 |
| 6’’ | 18.07 | 18.05 | 17.8 | 18.03 | 18.33 |
| 1’’’ | 98.73 | 98.54 | 98.5 | 98.65 | 99.01 |
| 2’’’ | 37.12 | 36.24 | 36.2 | 36.09 | 38.65 |
| 3’’’ | 68.33 | 69.33 | 69.4 | 69.52 | 67.00 |
| 4’’’ | 72.69 | 80.16 | 80.0 | 80.15 | 82.86 |
| 5’’’ | 69.73 | 69.33 | 69.3 | 68.11 | 69.05 |
| 6’’’ | 18.03 | 18.02 | 17.8 | 18.01 | 18.00 |
| COCH |  | 171.30  21.22 | 171.3  21.1 | 170.35  21.20 |  |
| 1’’’’ |  | 104.09 | 104.1 | 104.13 |  |
| 2’’’’ |  | 73.48 | 73.4 | 73.54 |  |
| 3’’’’ |  | 76.05 | 76.0 | 76.07 |  |
| 4’’’’ |  | 69.63 | 69.69 | 69.73 |  |
| 5’’’’ |  | 76.25 | 76.2 | 76.32 |  |
| 6’’’’ |  | 61.45 | 61.4 | 61.49 |  |
